# Endurance exercise attenuates juvenile irradiation-induced skeletal muscle functional decline and mitochondrial stress

**DOI:** 10.1186/s13395-022-00291-y

**Published:** 2022-04-12

**Authors:** Thomas N. O’Connor, Jacob G. Kallenbach, Haley M. Orciuoli, Nicole D. Paris, John F. Bachman, Carl J. Johnston, Eric Hernady, Jacqueline P. Williams, Robert T. Dirksen, Joe V. Chakkalakal

**Affiliations:** 1 Department of Biomedical Genetics, Genetics, Development and Stem Cells Graduate Program, University of Rochester Medical Center, Rochester, NY USA; 2 Department of Pharmacology and Physiology, University of Rochester Medical Center, Rochester, NY USA; 3 Department of Biomedical Engineering, University of Rochester Medical Center, Rochester, NY USA; 4 Department of Biology, Biological Sciences, University of Rochester, Rochester, NY USA; 5 Department of Pathology and Laboratory Medicine, Cell Biology of Disease Graduate Program, University of Rochester Medical Center, Rochester, NY USA; 6 Department of Pediatrics, University of Rochester Medical Center, Rochester, NY USA; 7 Department of Environmental Medicine, University of Rochester Medical Center, Rochester, NY USA; 8 Department of Orthopaedic Surgery and Cell Biology, Duke University, Durham, NC USA

**Keywords:** Radiation, Exercise, Muscle, Physiology, Calcium handling, Oxidative/nitrosative stress, Mitochondria

## Abstract

**Background:**

Radiotherapy is commonly used to treat childhood cancers and can have adverse effects on muscle function, but the underlying mechanisms have yet to be fully elucidated. We hypothesized that endurance exercise following radiation treatment would improve skeletal muscle function.

**Methods:**

We utilized the Small Animal Radiation Research Platform (SARRP) to irradiate juvenile male mice with a clinically relevant fractionated dose of 3× (every other day over 5 days) 8.2 Gy X-ray irradiation locally from the knee to footpad region of the right hindlimb. Mice were then singly housed for 1 month in cages equipped with either locked or free-spinning voluntary running wheels. Ex vivo muscle contractile function, RT-qPCR analyses, resting cytosolic and sarcoplasmic reticulum (SR) store Ca^2+^ levels, mitochondrial reactive oxygen species levels (MitoSOX), and immunohistochemical and biochemical analyses of muscle samples were conducted to assess the muscle pathology and the relative therapeutic impact of voluntary wheel running (VWR).

**Results:**

Irradiation reduced fast-twitch extensor digitorum longus (EDL) muscle-specific force by 27% compared to that of non-irradiated mice, while VWR post-irradiation improved muscle-specific force by 37%. Radiation treatment similarly reduced slow-twitch soleus muscle-specific force by 14% compared to that of non-irradiated mice, while VWR post-irradiation improved specific force by 18%. We assessed intracellular Ca^2+^ regulation, oxidative stress, and mitochondrial homeostasis as potential mechanisms of radiation-induced pathology and exercise-mediated rescue. We found a significant reduction in resting cytosolic Ca^2+^ concentration following irradiation in sedentary mice. Intriguingly, however, SR Ca^2+^ store content was increased in myofibers from irradiated mice post-VWR compared to mice that remained sedentary. We observed a 73% elevation in the overall protein oxidization in muscle post-irradiation, while VWR reduced protein nitrosylation by 35% and mitochondrial reactive oxygen species (ROS) production by 50%. Finally, we found that VWR significantly increased the expression of PGC1α at both the transcript and protein levels, consistent with an exercise-dependent increase in mitochondrial biogenesis.

**Conclusions:**

Juvenile irradiation stunted muscle development, disrupted proper Ca^2+^ handling, damaged mitochondria, and increased oxidative and nitrosative stress, paralleling significant deficits in muscle force production. Exercise mitigated aberrant Ca^2+^ handling, mitochondrial homeostasis, and increased oxidative and nitrosative stress in a manner that correlated with improved skeletal muscle function after radiation.

**Supplementary Information:**

The online version contains supplementary material available at 10.1186/s13395-022-00291-y.

## Background

Radiotherapy is the standard care for cancer patients, being used on roughly 50% of all patients at some point during treatment [1]. While radiation’s cytotoxic effects are intended to target transformed cancer cells, off-target effects have been observed that can lead to impaired muscle growth and function [2–5]. Radiation can be particularly disruptive to juvenile cancer patients as it primarily acts by damaging DNA and disrupting proper temporal gene expression that is necessary for proper development. Juvenile cancer survivors suffer from accelerated aging and experience muscle weakness and atrophy in the irradiated limb, decreasing quality of life [6–9]. Thus, understanding the mechanisms by which radiation disrupts proper skeletal muscle development and function could aid in improved treatment outcomes of the ever-growing cancer survivor population.

Ionizing radiation has a multitude of effects at the cellular level, most notably inducing damage to DNA, proteins, and lipids alike, which can lead to cell cycle arrest and cell death if left unresolved [10]. Mitochondrial DNA is even more vulnerable to damage induced by ionizing radiation due to the relatively less efficient damage repair mechanisms [11, 12]. Mitochondrial damage results in increased reactive oxygen species (ROS) production, mitophagy, and mitochondrial dysfunction, leading to disrupted ATP production and diminished muscle function [13, 14]. While low levels of oxidative stress play an important role in cell signaling [15, 16], excessive oxidative stress disrupts proper protein-protein interactions, alters gene expression, and impairs organelle and membrane integrity [15, 17]. Furthermore, juvenile irradiation significantly alters the expression of Ca^2+^-handling proteins that leads to disruption of both Ca^2+^-dependent cellular signaling processes and excitation-contraction coupling in the skeletal muscle [4].

Exercise, on the other hand, exhibits an array of beneficial effects on overall health, along with direct adaptive responses to the skeletal muscle itself. Exercise promotes protein translation, leading to increased turnover of damaged proteins and organelles [16, 18]. Similarly, prolonged exercise increases mitochondrial content in part through an upregulation of Pgc1α, the master regulator of mitochondrial biogenesis [15, 19–21]. Due to increased ATP demand with exercise, there is an increased production of ROS and reactive nitrogen species (RNS) as byproducts of mitochondrial respiration following sustained muscle activity [16]. However, exercise concomitantly upregulates the antioxidant response in the skeletal muscle to scavenge excess ROS/RNS [22, 23]. In addition to an upregulated antioxidant response, exercise promotes adaptations in the Ca^2+^ handling machinery to support efficient, repetitive muscle contractions [19]. One such adaptation includes alterations in spatial localization of proteins and organelles to allow for more efficient Ca^2+^ handling and signaling including the increased association of the mitochondria with calcium release units, improving mitochondrial Ca^2+^ uptake/function, and enhancing store-operated Ca^2+^ entry [19, 20, 22].

Here, we examined the effects of endurance exercise on skeletal muscle growth and function in mice subjected to juvenile fractionated irradiation. We further demonstrate that 1 month of endurance exercise mitigates muscle functional deficits following juvenile hindlimb irradiation. This was associated with corrections in calcium handling and oxidative/nitrosative stress.

## Methods

### Animals

This study was carried out in strict accordance with the recommendations in the Guide for the Care and Use of Laboratory Animals of the National Institutes of Health. All procedures involving animals were approved by the Institutional Animal Care and Use Committee (IACUC) at the University of Rochester called the University Committee on Animal Resources (UCAR). C57BL/6J juvenile/adult (1–3-month-old, Jackson Labs) male mice were used for all experiments and were singly housed at 8 weeks of age in accordance with the UCAR protocol. Mice were maintained on a 12:12 light/dark cycle and provided ad libitum access to pelleted feed and standard drinking water (Hydropac)

### SARRP radiation

All ionizing X-ray irradiation was delivered using the Small Animal Radiation Research Platform (SARRP, XStrahl) with a 10 × 25 mm variable collimator as previously described [3, 4]. Mice were anesthetized with vaporized isoflurane. Fractionated irradiation was administered to 4-week-old mice using 3 fractions of 8.2-Gy radiation delivered on Monday, Wednesday, and Friday. Radiation was delivered locally to the lower right hindlimb from the footpad to the tibial plateau.

### Voluntary wheel running exercise

Low-profile wireless rodent wheels (ENV-047 wheels, Med Associates, Fairfax, VT, USA) were used in singly housed mouse cages to track chronic endurance exercise running activity over a 4-week period, with sedentary control animals placed in cages having wheels that were locked and unable to rotate. Wheels were connected to a wireless central hub that recorded running activity every 30 s in the Wheel Manager software, with the Wheel Analysis software (Med Associates) used to report data as the total distance run each day (km/day). The percentage of daytime running activity was reported as the total running activity that was conducted during the 12 h daily that the lights were on. Once mice were removed from the wheels, terminal experiments were performed immediately thereafter.

### Ex vivo muscle force generation assessment

Muscle force generation capacity was analyzed for EDL and soleus muscles using the Aurora Scientific (ASI) muscle contraction system [21, 24, 25]. Briefly, mice were anesthetized with vaporized isoflurane, the tibialis anterior (TA) muscle removed, and then the proximal and distal tendons of the EDL or soleus muscles were sutured and removed. The muscles were adjusted to their optimal length (Lo), electrically stimulated at increasing frequencies, subjected to three 150-Hz warm-up stimulations, and then finally assessed for force generation capacity. Muscle force data were recorded and analyzed with the Dynamic Muscle Control (DMC) and Analysis (DMA) software. Physiologic cross-sectional area (P-CSA) was calculated as (muscle weight [mg])/(1.056 × (0.44 or 0.71) × length [mm]), where 1.056 = muscle density [g/cm^3^], 0.44 = EDL angular factor, and 0.71 = soleus angular factor [26].

### RNA extraction and RT-qPCR

RNA isolation and RT-qPCR were performed as previously described [21]. Briefly, the gastrocnemius muscles were removed, flash-frozen in TRIzol Reagent (Life Technologies), homogenized, and RNA isolated using RNeasy Plus Mini Kit (Qiagen) according to the manufacturer’s protocols. Then, cDNA was synthesized using the qScript cDNA SuperMix (QuantaBio). RT-qPCR was performed on a Step One Plus Real-Time PCR machine (Applied Biosystems) using SYBR Green FastMix (QuantaBio). Transcript levels from each experiment were standardized to their internal *Gapdh* gene expression and then normalized to the control condition.

For qPCR, we used the following primers:

- Dnm1l forward primer 5′-TTACGGTTCCCTAAACTTCACG-3′
- Dnm1l reverse primer 5′-GTCACGGGCAACCTTTTACGA-3′
- Fis1 forward primer 5′-TGTCCAAGAGCACGCAATTTG-3′
- Fis1 reverse primer 5′-CCTCGCACATACTTTAGAGCCTT-3′
- Mfn1 forward primer 5′-CCTACTGCTCCTTCTAACCCA-3′
- Mfn1 reverse primer 5′-AGGGACGCCAATCCTGTGA-3′
- Pgc1α forward primer 5′-TATGGAGTGACATAGAGTG-3′
- Pgc1α reverse primer 5′-CCACTTCAATCCACCCAGAAAG-3′

### Tissue sectioning and immunostaining

The muscles were removed, placed in 30% sucrose overnight at 4 °C, embedded in OCT (Tissue Tek), flash-frozen using dry ice-cooled isopentane, stored at − 80 °C, and sectioned at 10 μm thickness. Prior to immunostaining, tissue sections were fixed in 4% PFA (except muscle fiber type sections), permeabilized with PBS-T (0.2% Triton-x-100 in PBS) for 10 min, and blocked in 10% normal goat serum (NGS, Jackson ImmunoResearch) for 30 min at room temperature (RT), then primary antibodies were applied. If using mouse primary antibodies, sections were blocked in 3% AffiniPure Fab fragment goat anti-mouse (Jackson ImmunoResearch) with 2% NGS at RT for 1 h. Primary antibody incubation in 2% NGS/PBS was performed for 2 h at RT or overnight at 4 °C followed by secondary antibody incubation for 1 h at RT. DAPI staining was performed to identify the nuclei. All slides were mounted with Fluoromount-G (SouthernBiotech). The sections were imaged at × 4, × 10, and × 20 magnifications on the Echo Revolve microscope and analyzed using ImageJ (NIH). Sample analyses were performed by investigators blinded to the experimental group.

### Single FDB myofiber isolation

All resting cytosolic, SR store Ca^2+^, and MitoSOX experiments were conducted using single, acutely dissociated flexor digitorum brevis (FDB) myofibers, as previously described [19]. FDB muscles were dissected from the hind limb footpads and placed in Ringer’s solution (145 mM NaCl, 5 mM KCl, 2 mM CaCl_2_, 1 mM MgCl_2_, 10 mM HEPES, pH 7.4) supplemented with 1 mg/mL collagenase A (Roche Diagnostics, Indianapolis, IN, USA) while rocking gently at 37 °C for 1 h. FDB myofibers were then liberated on glass-bottom dishes by gentle trituration in Ringer’s solution using three sequentially increasing gauge glass pipettes and then allowed to settle for 20 min. Only healthy fibers with clear striations and no observable damage were used for experiments.

### Resting Ca^2+^ measurements

Resting free cytosolic Ca^2+^ concentration was determined as previously described [19]. Briefly, isolated FDB myofibers were loaded with 4 μM fura-2 AM (Thermo Fisher, Carlsbad, CA, USA) in Ringer’s solution at RT for 30 min followed by a 30-min washout in dye-free Ringer’s solution. Loaded fibers were placed on the stage of an inverted epifluorescence microscope (Nikon Instruments) and alternatively excited at 340 and 380 nm (20 ms exposure per wavelength, 2 × 2 binning) using a monochromator-based illumination system with fluorescence emission at 510 nm captured using a high-speed QE CCD camera (TILL Photonics, Graefelfing, Germany). 340/380 ratios from cytosolic areas of interest were calculated using TILL vision software (TILL Photonics Graefelfing, Germany), analyzed using ImageJ and converted to resting free Ca^2+^ concentrations using a fura-2 calibration curve approach described previously [27].

### Total releasable SR Ca^2+^ store content measurements

Total Ca^2+^ store content was determined as previously described [19]. Briefly, FDB myofibers were loaded with 5 μM fura-FF AM (AAT Bioquest, Sunnyvale, CA, USA), a low-affinity ratiometric Ca^2+^ dye, at RT for 30 min, followed by a 30-min washout in dye-free Ringer’s. Total releasable SR Ca^2+^ store content was calculated from the peak change in the fura-FF ratio (ΔRatio_340/380_) upon application of ICE Ca^2+^ release cocktail (10 μM ionomycin, 30 μM cyclopiazonic acid, and 100 μM EGTA) in Ca^2+^-free Ringer’s solution. Peak change in the fura-FF ratio was calculated using Clampfit 10.0 (Molecular Devices, Sunnyvale, CA, USA).

### Mitochondrial ROS production

Mitochondrial ROS production was assessed using a procedure modified from Lee et al. [28]. Briefly, single FDB myofibers were incubated with 5 μM MitoSOX Red (Thermo Fisher) in Tyrode’s solution (121 mM NaCl, 5 mM KCl, 1.8 mM CaCl_2_, 500 μM MgCl_2_, 400 μM NaH_2_PO_4_, 5.5 mM glucose, 24 mM NaHCO_3_, 100 μM EDTA) for 10 min at RT, followed by incubation in dye-free Tyrode’s solution for 10 min at RT. Loaded myofibers were excited at 488 nm, and emission was captured at 605 nm. Images were taken after the 10-min dye-free incubation and 10 min after taking the initial image. Images were taken using a Nikon Digital Eclipse C1 confocal microscope using a × 40 objective, and image analysis was done using the EZ-C1 software and ImageJ. Change in fluorescence after 10 min was normalized to the original measurement and reported as Δ*f*/*f*_0_.

### Western blot analyses

TA and soleus muscles were flash-frozen in liquid nitrogen and stored at − 80 °C until ready for use. The muscles were mechanically homogenized in RIPA lysis buffer (20 mM Tris-HCl pH 7.5, 150 mM NaCl, 1 mM Na_2_EDTA, 1 mM EGTA, 1% NP-40, 1% sodium deoxycholate, 1 mM Na_3_VO_4_, 10 mM NaF) and supplemented with Halt protease inhibitor, as recommended by the manufacturer. Samples were centrifuged at 13,000*g* for 30 min; supernatants were retained and then protein concentration was determined using the Bio-Rad DC assay (500-0116). Ten micrograms of total protein was separated on 12% sodium dodecyl sulfate-polyacrylamide gel electrophoresis (SDS-PAGE) and transferred to a nitrocellulose membrane. The membranes were briefly stained with a 0.1% Ponceau S solution (Sigma-Aldrich, P3504) to ensure equal protein loading. The membranes were probed with primary antibodies for 2 h at RT or overnight at 4 °C while shaking, diluted in TBS-T (20 mM Tris, 150 mM NaCl, 0.1% Tween 20, pH 7.6, 3% bovine serum albumin). Secondary antibodies were applied, diluted in TBS-T supplemented with 5% non-fat dry milk for 1 h at RT while shaking. Blots were imaged on a LI-COR Odyssey gel imaging system, and band intensity was quantified using Image Studio Lite (LI-COR Biosciences, Lincoln, NE, USA) using GAPDH as a loading control for each sample. Analysis for 3-NT and oxyblot data was conducted by assessing the overall intensity of the entire lane.

### Oxyblot for oxidized proteins

Oxidized proteins were quantified by the Oxyblot Protein Oxidation Kit (Millipore) using 10 μg of muscle lysate for each sample following the manufacturer’s protocol.

### Antibodies

The following antibodies were used: rat anti-laminin-α2 (1:1500, Sigma-Aldrich, L0663), DAPI (1:3000), mouse anti-BA-D5 (MyHC-I, IgG2b, 1:40, Developmental Studies Hybridoma Bank (DSHB)), mouse anti-SC-71 (MyHC-IIA, IgG1, 1:40, DSHB), mouse anti-BF-F3 (MyHC-IIB, IgM, 1:40, DSHB), rabbit anti-PGC1α (1:1000, Novus Biologicals), rabbit ant-MCU (1:1000, Cell Signaling, D2Z3B), mouse anti-PMCA (1:2000, Thermo Fisher, 5F10), mouse anti-NCX (1:500, Swant, R3F1), mouse anti-MFN1 (1:500, Neuromab, 75-162), mouse anti-GAPDH (1:50,000, Thermo Fisher, AM4300), mouse anti-CASQ1 (1:5000, Affinity BioReagents, MA3-913), rabbit anti-pan SERCA (1:10,000, Santa Cruz, sc-30110), rabbit anti-VDAC (1:5000, Sigma Aldrich), rabbit anti-DNP (1:150, Sigma Aldrich), mouse anti-3-NT (1:1000, Sigma Aldrich, N5538), Alexa Fluor 405-conjugated goat anti-mouse IgG2b (1:1500, Thermo Fisher, A-21141), Alexa Fluor 488-conjugated goat anti-mouse IgM (1:1500, Thermo Fisher, A-21042), Alexa Fluor 594-conjugated goat anti-mouse IgG1 (1:1500, Thermo Fisher, A-21125), AlexaFluor 488-conjugated goat anti-mouse IgG (1:1500, Thermo Fisher, A-11001), AlexaFluor 488-conjugated goat anti-rabbit IgG (1:1500, Thermo Fisher, A-11034), AlexaFluor 647-conjugated goat anti-rat IgG (1:1500, Thermo Fisher, A-21247), goat anti-rabbit IRDye800 (1:10000, LiCor), goat anti-mouse IRDye800 (1:10,000, LiCor), and goat anti-mouse IRDye700 (1:10,000, LiCor).

### Statistical analyses

Statistical calculations were performed using the GraphPad Prism 9 software. Statistical significance was determined through Student’s *t*-tests with Welch’s corrections (unpaired, two-tailed, 95% confidence interval) or ANOVA (one-way or two-way, followed by Tukey multiple comparisons test), where *p* < 0.05 was considered statistically significant (*^/#^*p* < 0.05, **^/##^*p* < 0.01, ***^/###^*p* < 0.001, ****^/####^*p* < 0.0001). Error bars are represented as ± standard error of the mean (SEM).

## Results

### Voluntary wheel running attenuates juvenile irradiation-induced skeletal muscle functional decline

**Fig. 1:**
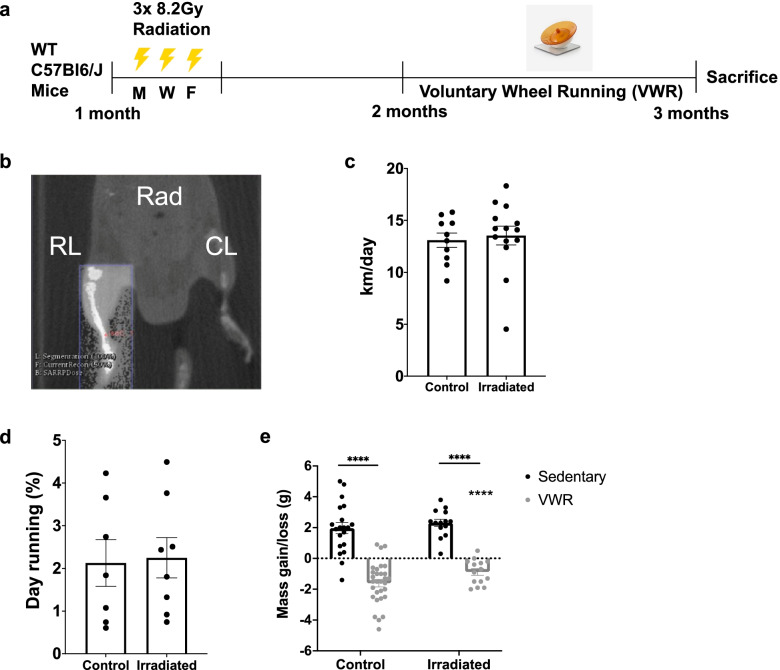
Juvenile mice display similar activity and body mass changes following 1-month VWR exercise despite irradiation. **a** Experimental design of juvenile fractionated radiation 3× 8.2 Gy, (M, W, F) on 1-month-old WT C57Bl6/j mice followed by 1 month of voluntary wheel running (VWR) exercise at 2 months of age. Mice were sacrificed at 3 months of age. Non-irradiated mice and mice unable to engage with the running wheel were used as radiation and exercise controls, respectively. **b** Small Animal Radiation Research Platform (SARRP) image displaying area of localized X-ray irradiation. **c** Running activity of irradiated and control non-irradiated mice. Each dot is representative of one mouse. **d** Percent day running of irradiated and control non-irradiated mice. Each dot represents one mouse. **e** Change in body mass (g) following 1-month VWR exercise. Each dot is representative of one mouse. Two-way ANOVA with multiple comparisons ****p < 0.0001. Isolated asterisks denote the ANOVA group effect of exercise. Data is displayed as mean ± s.e.m

To assess the consequences of voluntary running on muscle function following juvenile irradiation, one hindlimb from 4-week-old prepubertal mice received 3 doses of local 8.2-Gy radiation every other day over 5 days (MWF) (Fig. 1b). After treatment, mice were singly housed and allowed to engage in 1 month of voluntary wheel running (VWR) beginning at 8 weeks of age (Fig. 1a). Singly housed mice with locked wheels served as sedentary controls. Regardless of the radiation treatment, mouse running activity during the 1-month period was similar between juvenile irradiated and non-irradiated mice (Fig. 1c). Furthermore, no difference in the circadian patterns of running activity was observed (Fig. 1d). Finally, both juvenile irradiated and non-irradiated mice exhibited similar levels of net body mass gain/loss under sedentary/VWR conditions, respectively (Fig. 1e). Thus, local juvenile irradiation to a hindlimb does not hinder the ability of mice to engage in VWR activity.

**Fig. 2:**
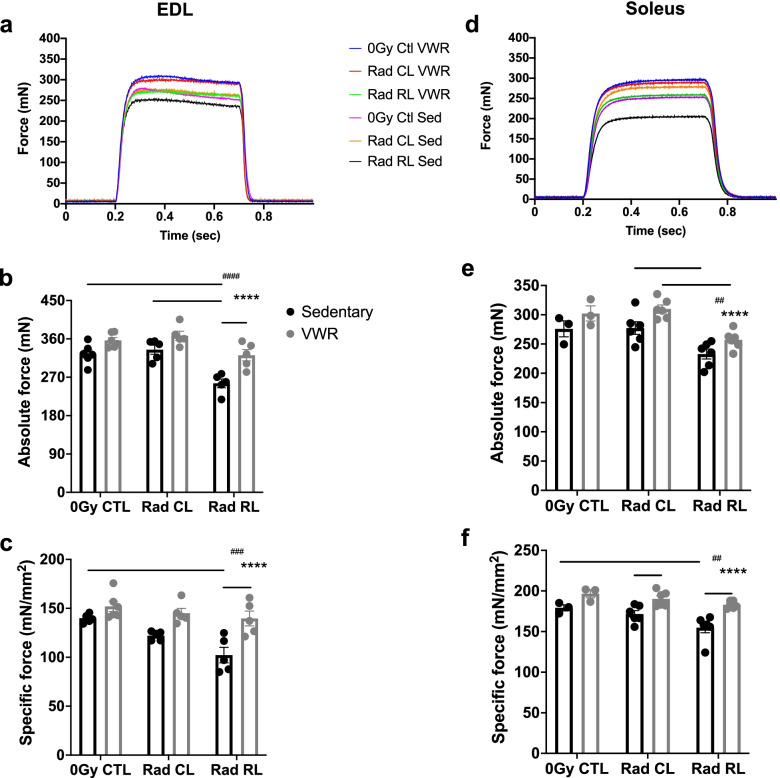
Exercise post-juvenile irradiation attenuates muscle force generation capacity deficits. EDL (left) and soleus (right) ex vivo muscle physiology force readouts. **a**, **d** Representative 500 ms 150 Hz stimulation muscle force (mN) trace. **b**, **e** Maximal absolute force (mN) generated at 150 Hz stimulation. **c**, **f** Maximal specific force (mN/mm^2^) generated at 150 Hz stimulation. *n* = 3–7 mice per condition. Two-way ANOVA with multiple comparisons, *p < 0.05, **^/##^*p* < 0.01, ***^/###^*p* < 0.001, ****^/####^*p* < 0.0001. Isolated asterisks denote ANOVA group effect of exercise, and isolated pound signs denote ANOVA group effect of radiation. Data displayed as mean ± s.e.m

To examine the effects of VWR activity on juvenile irradiated skeletal muscle function, fast-contracting extensor digitorum longus (EDL) and slow-contracting soleus muscles were excised and processed to assess ex vivo force generation and contractile kinetics (Fig. 2a, d, Supplemental Fig. 1). Upon maximal electrical stimulation, juvenile irradiated (Rad RL) EDL and soleus muscles from sedentary mice displayed deficits in absolute (Fig. 2b) and specific force (Fig. 2c) in comparison with control contralateral non-irradiated (Rad CL) muscles and muscles from non-irradiated (0Gy CTL) sedentary mice. Importantly, 1 month of VWR activity significantly attenuated juvenile irradiation-induced EDL absolute force deficits and restored specific force capacity to control levels. Although 1 month of VWR activity did not significantly alter Rad RL soleus muscle absolute force generation capacity (Fig. 2e), it did attenuate juvenile irradiation-induced-specific force deficits (Fig. 2f). Consistent with previous studies, juvenile irradiation led to decreased EDL and soleus muscle mass and individual myofiber cross-sectional area (CSA) (Supplemental Fig. 2) [3–5]. However, VWR activity did not significantly increase Rad RL raw muscle weights or myofiber CSA (Supplemental Fig. 2a, b). Thus, VWR activity attenuated juvenile irradiation-induced-specific force deficits regardless of muscle type and independent of any increase in muscle mass or myofiber size.

**Fig. 3:**
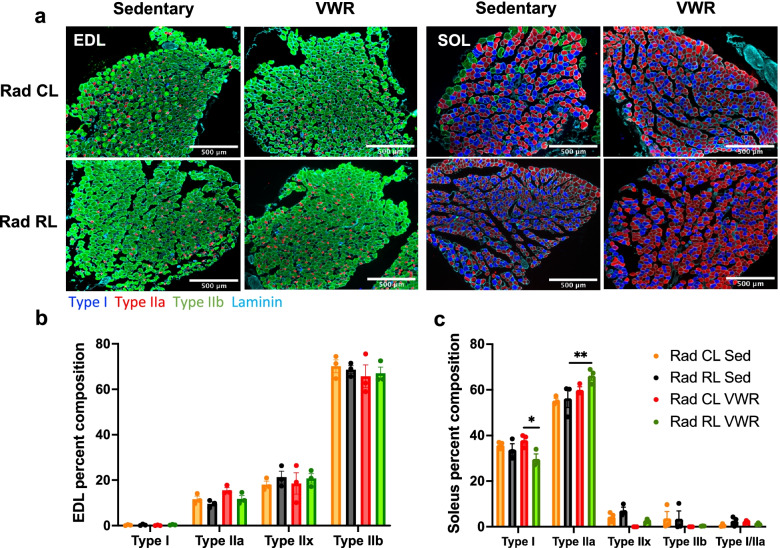
Juvenile irradiated exercised soleus muscle displays a minor shift from type I to type IIa muscle fibers. Muscle fiber type analysis of EDL and soleus muscle from 3-month-old WT irradiated (Rad RL) and non-irradiated (Rad CL) mice after 1 month of VWR exercise (running) or sedentary control. **a** Muscle cross-sections from sedentary (left) and VWR exercised (right), non-irradiated contralateral (Rad CL) (top), and irradiated limb (Rad RL) (bottom) muscle. EDL sections (left) are labeled as such and appear primarily green with soleus sections (right) appearing primarily blue and red. Sections stained for type I (blue), type IIa (red), type IIb (green), and laminin (teal). Type IIx fibers are unstained and appear black. **b** Quantified fiber type distribution of muscle cross-sections from **a** for the **b** EDL and **c** soleus muscles. *n* = 3 mice for each condition. Two-way ANOVA with multiple comparisons. *p < 0.05, **p < 0.01. A significant interaction between variables was observed for soleus muscle fiber type data, *p* = 0.0006. Data displayed as mean ± s.e.m

Next, the consequences of juvenile irradiation and VWR activity on various muscle contractile parameters were evaluated. Juvenile irradiation led to a reduction in muscle impulse (area under the curve, AUC) and specific impulse. Although VWR activity restored Rad RL EDL impulse and specific impulse values to control levels, only specific impulse was improved in Rad RL soleus muscles (Supplemental Fig. 1c, d, i, j). The maximum rate of force production (activation) of Rad RL EDL and soleus muscle upon stimulation was significantly reduced as compared to Rad CL and/or 0Gy CTL muscles and VWR activity exhibited a significant group effect on increasing the rate of muscle activation across all conditions (Supplemental Fig. 1e, k). Juvenile irradiation led to a significant reduction in the maximum rate of force relaxation of both EDL and soleus muscles that was increased in response to VWR activity (Supplemental Fig. 1f, l). Finally, we assessed muscle fiber type distribution in the EDL and soleus muscles (Fig. 3a) to determine the effect of radiation and exercise on fiber type plasticity. We did not find any significant fiber type shift in the EDL muscle (Fig. 3b); however, a modest, albeit significant, shift from type I to type IIa muscle fibers was observed in Rad RL soleus muscles following VWR (Fig. 3c).

### Exercise increases Ca^2+^ store content in irradiated muscle

**Fig. 4:**
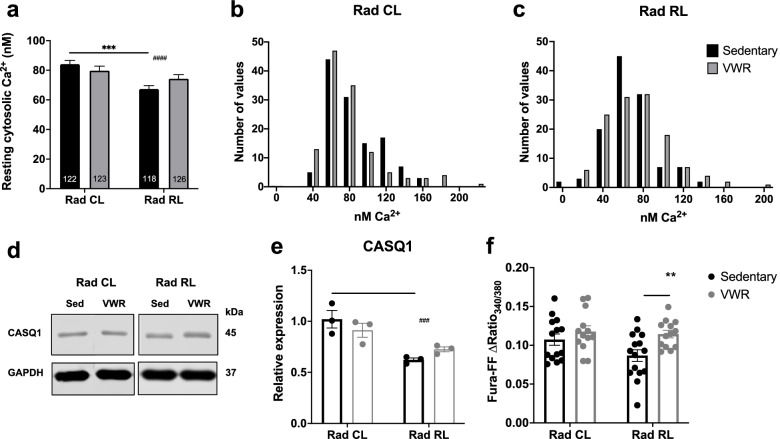
Exercise increases Ca^2+^ store content in irradiated muscle. Ca^2+^ analysis from 3-month-old WT mouse irradiated (Rad RL) and non-irradiated (Rad CL) muscle after 1 month of VWR exercise (running) or sedentary control. **a** Resting cytosolic Ca^2+^ measurements from fura-2-loaded single FDB myofibers. Biological *n* = 9 mice per condition, technical *n* value displayed directly on the graph as the number of myofibers measured per condition. **b** Single myofiber cytosolic resting Ca^2+^ concentration frequency distribution for non-irradiated contralateral (Rad CL) and **c** irradiated (Rad RL) FDB myofibers. **d** TA whole muscle protein lysate immunoblot of non-irradiated (Rad CL) and irradiated (Rad RL) sedentary and exercised muscle. **e** Quantification of calsequestrin1 protein expression from **d** as normalized to GAPDH. *N* = 3 mice per condition. **f** Total SR store Ca^2+^ content measurements from isolated non-irradiated and irradiated single FDB myofibers loaded with fura-FF and perfused with ICE store-depleting solution from sedentary and exercised mice. Biological *n* = 4–5 mice per condition, technical *n* value displayed directly as individual data points on graph as the number of myofibers measured per condition. Two-way ANOVA with multiple comparisons *p < 0.05, **p < 0.01, ***^/###^*p* < 0.001, ^####^*p* < 0.0001. Isolated asterisks denote the ANOVA group effect of exercise. Isolated pound signs denote the ANOVA group effect of radiation. A significant interaction between variables was observed for the analysis of resting cytosolic Ca^2+^, *p* = 0.0375. Data displayed as mean ± s.e.m

In the skeletal muscle, regulation of Ca^2+^ handling is critical for excitation-contraction coupling, which is used to drive myofilament cross-bridge cycling and muscle contraction [29, 30]. Furthermore, dysregulated Ca^2+^ handling is associated with prolonged injury responses, such as elevated oxidative stress, that can negatively impact muscle function [31]. We found that myofibers from juvenile irradiated mice displayed a significant reduction in resting cytosolic Ca^2+^ concentration (Fig. 4a–c). Importantly, no significant reduction in resting cytosolic Ca^2+^ was observed between Rad CL and Rad RL myofibers following 1 month of VWR. The expression of calsequestrin-1 (CASQ1), the primary SR Ca^2+^ binding protein in skeletal muscle, was significantly reduced in the irradiated muscle (Fig. 4d, e). We also observed a significant reduction in the expression of the sarco-endoplasmic reticulum Ca^2+^ ATPase (SERCA) pump in Rad RL sedentary muscle (Supplemental Fig. 3d, e). However, 1 month of VWR increased total SR Ca^2+^ store content (Fig. 4d) despite decreasing SERCA expression and causing no significant changes in CASQ1 expression. Together, these results show that the functional decline observed in Rad RL muscle correlates with a disruption in normal Ca^2+^ handling and that 1 month of VWR activity potentially mitigates these effects by adapting Ca^2+^ handling.

### Nitrosative and mitochondrial oxidative stress in irradiated muscle is reduced with exercise

**Fig. 5:**
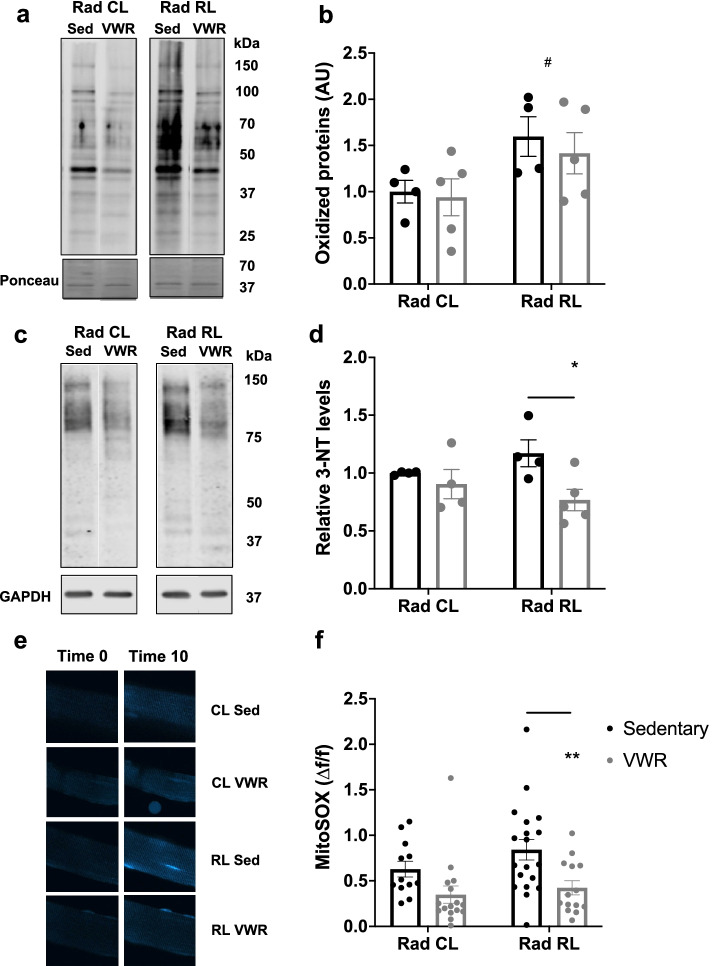
Nitrosative and mitochondrial oxidative stress in irradiated muscle is reduced with exercise. **a** Soleus whole muscle protein lysate oxyblot from non-irradiated (Rad CL) and irradiated (Rad RL) muscle in sedentary and exercised mice. **b** Quantification of oxidized proteins from **a** normalized to ponceau stain and non-irradiated (Rad CL) sedentary muscle. *N* = 4–5 mice per condition. **c** Soleus whole muscle protein lysate 3-nitrotyrosine (3-NT) immunoblot from non-irradiated (Rad CL) and irradiated (Rad RL) muscle in sedentary and exercised mice. **d** Quantification of 3-NT levels normalized to GAPDH and non-irradiated sedentary controls from **c** immunoblot. *N* = 4–5 mice per condition. **e** Images of MitoSOX Red-loaded FDB myofibers from non-irradiated (Rad CL) and irradiated (Rad RL) muscle in sedentary and exercised mice at time 0 and time 10 (min). **f** Quantification of MitoSOX fluorescence from **e**. Technical *n* is displayed directly on the graph, biological *n* = 5 mice per condition. Two-way ANOVA with multiple comparisons. *^/#^*p* < 0.05, **p < 0.01. Isolated asterisks denote the ANOVA group effect of exercise. Isolated pound signs denote the ANOVA group effect of radiation. Data displayed as mean ± s.e.m

Several muscle disorders that result from Ca^2+^ dysregulation, including central core disease (CCD) and malignant hyperthermia (MH), lead to increased oxidative stress and mitochondrial dysfunction [32–34]. Similarly, ionizing radiation increases the production of reactive oxygen and nitrogen species (ROS/RNS) in the skeletal muscle, both acutely via hydrolysis and chronically as a result of production by damaged organelles, particularly mitochondria [12, 35–38]. In addition, exercise upregulates antioxidant pathways to enhance the scavenging of ROS produced from repetitive muscle contraction, thus preserving proper signaling, Ca^2+^ regulation, and overall muscle function [22, 35, 39, 40]. To this end, we determined the impact of VWR on enhanced ROS/RNS production and overall oxidative/nitrosative stress in irradiated skeletal muscle. Oxyblot analyses of whole soleus muscle lysates revealed an elevation in the overall protein oxidation following juvenile irradiation that was not exacerbated with VWR activity (Fig. 5a, b). To assess nitrosative stress, we evaluated levels of 3-nitrotyrosine (3-NT), a product of tyrosine nitration mediated by RNS. We observed significantly elevated 3-NT levels in Rad RL sedentary muscle that were reduced following VWR (Fig. 5c, d). Finally, we used a mitochondrial ROS sensor, MitoSOX Red, to directly assess the rate of mitochondrial ROS production in acutely dissociated flexor digitorum brevis (FDB) myofibers from control and irradiated mice [41]. Consistent with an exercise-induced increase in ROS detoxification, the maximum rate of ROS production was significantly reduced in FDB myofibers from Rad RL mice after 1 month of VWR (Fig. 5e, f). These findings suggest that exercise may improve muscle function after juvenile irradiation through the combined effect of reducing cellular RNS and mitochondrial ROS levels.

### Exercise adapts the mitochondrial gene expression profile of irradiated muscle

**Fig. 6:**
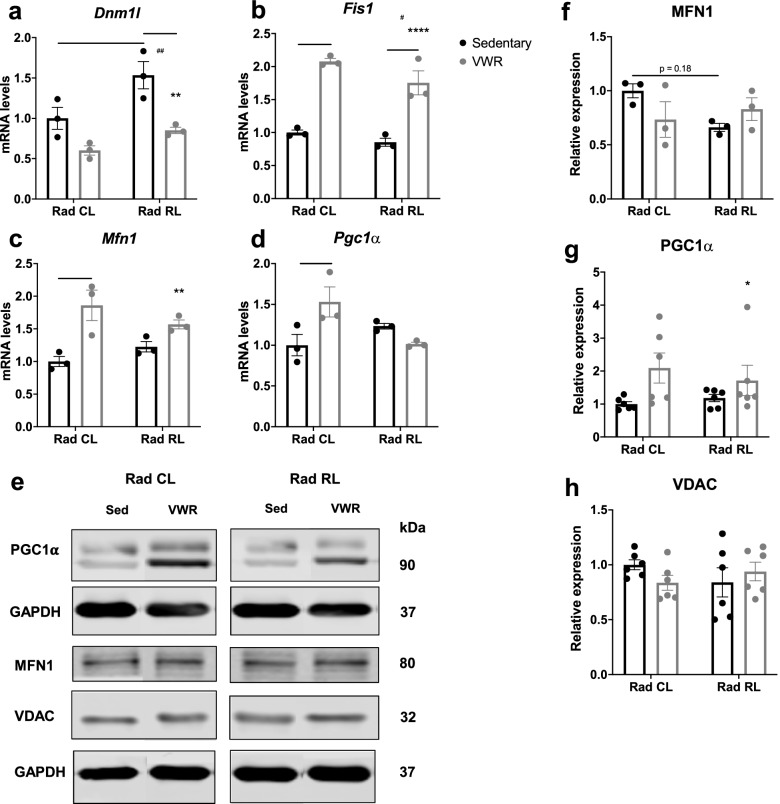
Exercise adapts the mitochondrial gene expression profile of irradiated muscle. **a** Whole muscle mRNA expression from 3-month-old WT mouse irradiated (Rad RL) and non-irradiated (Rad CL) muscle after 1 month of VWR exercise (running) or sedentary control for *Dnm1l*, **b***Fis1*, **c***Mfn1*, and **d***Pgc1α*. mRNA levels are reported as fold change relative to *Gapdh* and normalized to sedentary non-irradiated control. *N* = 3 mice for each condition. **e** TA whole muscle protein lysate immunoblot for PGC1α, MFN1, and VDAC. Quantification of **f** MFN1, **g** PGC1a, and **h** VDAC protein levels, as displayed in the immunoblot from TA lysate I, normalized to GAPDH and non-irradiated sedentary controls. *n* = 3 mice per condition except PGC1α and VDAC, *n* = 6. Two-way ANOVA with multiple comparisons. *^/#^*p* < 0.05, **^/##^*p* < 0.01, ***p < 0.001, ****p < 0.0001. Isolated asterisks denote the ANOVA group effect of exercise. Isolated pound signs denote the ANOVA group effect of radiation. A significant interaction between variables was observed in the analysis of *Pgc1α* transcript levels in **d**, *p* = 0.0113. Data displayed as mean ± s.e.m

The above findings raised the question as to the mechanism(s) by which VWR mitigates increased mitochondrial ROS production following irradiation. As previously noted, radiation causes mitochondrial damage, while exercise induces signaling processes that adapt mitochondrial dynamics and promote mitochondrial biogenesis [16, 39, 42–45]. We first assessed signaling associated with mitochondrial fission and breakdown by assessing mRNA levels of dynamin 1 like (*Dnm1l*) and mitochondrial fission 1 (*Fis1*). We observed elevated expression of *Dnm1l* transcripts in Rad RL sedentary muscle (Fig. 6a), as well as elevated expression of *Fis1* transcripts in Rad CL and RL muscle from mice after VWR (Fig. 6b). To assess signaling associated with mitochondrial fusion and biogenesis, we quantified mitofusin 1 (*Mfn1*) and PPARgamma coactivator 1-alpha (*Pgc1α*) transcript levels. Both transcripts were significantly increased only in Rad CL muscle from mice after VWR (Fig. 6c, d). Finally, we assessed the protein expression levels of MFN1, PGC1α, and voltage-dependent anion channel (VDAC), with VDAC levels serving as a readout of total mitochondrial content (Fig. 6e–h). While *Mfn1* transcript levels were elevated following 1 month of VWR, no differences at the protein level were observed following irradiation with or without 1 month of VWR (Fig. 6f). Although no significant changes were observed in VDAC expression (Fig. 6h), PGC1α expression was significantly increased after 1 month of VWR (Fig. 6g). Interestingly, the expression of the mitochondrial Ca^2+^ uniporter (MCU), which is responsible for mitochondrial Ca^2+^ uptake in the muscle, was reduced after VWR (Supplemental Fig. 3d, f). This observed reduction in MCU expression after VWR could reflect a muscle adaptation designed to limit oxidative stress levels since increased mitochondrial Ca^2+^ uptake is associated with increased mitochondrial ROS production [46]. Taken together, these data are consistent with VWR driving an altered gene expression signature designed to adapt mitochondrial dynamics and biogenesis to reduce ROS production and improve muscle function in juvenile irradiated muscle.

## Discussion

Here, we evaluated the feasibility of exercise as a potential therapeutic intervention to restore reduced muscle function post-juvenile irradiation. Based on previous findings in the field, ionizing radiation causes muscle atrophy, progenitor cell cycle arrest and death, and persistent inflammatory signaling [3–5, 21, 36]. We found that 1 month of VWR exercise improved specific force generation capacity regardless of muscle type that was associated with adapted Ca^2+^ handling as well as reduced oxidative/nitrosative stress.

Excitation-contraction coupling is the process by which an electrical signal in the transverse tubule membrane (i.e., action potential) is converted into Ca^2+^ release used to drive myofilament cross-bridge cycling that underlies muscle force production [47]. Dysfunctions in Ca^2+^ handling in the skeletal muscle underlie several muscle disorders including MH, CCD, and tubular aggregate myopathy (TAM) [29, 48, 49]. Our lab previously reported RNA sequencing (RNAseq) data that showed a significant reduction in the expression of several key Ca^2+^ handling genes (including CASQ1, CACNB1, CACNG1, and TNNT3) following juvenile irradiation [4]. Consistent with this, we found a significant reduction in CASQ1 protein expression in the muscle from sedentary Rad RL mice. As CASQ1 polymerization increases Ca^2+^ binding sites within the SR lumen, a reduction in CASQ1 expression could partly explain the reduced peak specific force production observed in Rad RL muscle from sedentary mice. Similarly, reduced SERCA expression in Rad RL muscle from sedentary mice would be expected to contribute to the observed reduction in the maximum rate of contractile relaxation. Resting cytosolic Ca^2+^ levels in the muscle are under tight regulatory control in order to maintain proper proteostasis and muscle tone, in addition to modulating a multitude of other Ca^2+^-dependent cellular processes. We found that free resting cytosolic Ca^2+^ concentration was reduced in Rad RL myofibers from sedentary mice. The mechanism for this reduction in resting Ca^2+^ concentration is unclear. We quantified the expression of plasma membrane Ca^2+^ ATPase (PMCA) pump and Na^+^-Ca^2+^ exchanger (NCX) as potential mechanisms for this reduction of cytosolic Ca^2+^. However, both PMCA and NCX expression were increased after VWR, consistent with an adaptive response to exercise designed to enhance Ca^2+^ efflux. Adding to the complexity surrounding intracellular Ca^2+^ handling, despite an increase in total SR Ca^2+^ store content, SERCA expression was reduced after VWR. Alternatively, the enhanced Ca^2+^ store content observed following VWR could reflect an increase in store-operated Ca^2+^ entry following exercise-dependent remodeling of specialized junctions between SR and transverse tubule membranes within the I band (referred to as “Ca^2+^ entry units”) [19, 22, 50].

Ca^2+^ also plays a critical role in regulating mitochondrial function. Mitochondrial Ca^2+^ uptake can depolarize the mitochondrial potential, stimulate enzymes of the tricarboxylic acid cycle, and promote ATP production [51], while excessive accumulation of mitochondrial Ca^2+^ can increase ROS production and trigger the opening of the mitochondrial permeability transition pore, ultimately resulting in mitochondrial swelling and cell death [48, 52]. We found a significant reduction in MCU expression 1 month after VWR, which could reflect a protective mechanism designed to limit mitochondrial Ca^2+^ overload, ROS production, and mitochondrial swelling/damage. This observation supports prior work showing that VWR exercise promotes mitochondrial coupling to the Ca^2+^ release unit (triad) and lowers oxidative stress in the muscle [22]. Ionizing radiation directly promotes mitochondrial DNA damage, as well as structural damage to the inner and outer mitochondrial membranes, thus compromising organelle integrity [14]. These alterations can lead to increased production of ROS from damaged mitochondria, perpetuating the radiation-induced ROS imbalance [53]. Elevated ROS/RNS levels also enhance Ca^2+^ leak from the SR by increasing the open probability of the ryanodine receptor, the SR Ca^2+^ release channel, leading to a reduction in SR Ca^2+^ content [48, 54, 55]. We found that the mitochondria in myofibers from irradiated sedentary juvenile mice produced significantly more ROS than mitochondria in myofibers from irradiated mice after 1 month of VWR exercise. An increase in oxidative and nitrosative stress was also observed in whole muscles as well, as muscle lysates from irradiated sedentary hindlimbs exhibited increased levels of oxidized and 3-NT proteins compared to that observed for muscles from irradiated mice after 1 month of VWR.

As exercise stimulates mitochondrial biogenesis, we probed the effect of VWR exercise on the potential removal of damaged, high ROS-producing mitochondria post-juvenile irradiation [18, 22]. By quantifying the levels of transcripts and proteins associated with mitochondrial fission (Dnm1l, Fis1), fusion (Mfn1), and biogenesis (Pgc1a), we observed a gene expression signature consistent with adapted mitochondrial dynamics and improved homeostasis in the muscle from irradiated mice after VWR. On the other hand, irradiated muscle from sedentary juvenile mice displayed a gene expression profile indicative of a higher propensity toward an increased mitochondrial breakdown in the absence of mitochondrial biogenesis. These observations are consistent with our observation that mitochondria from mice after VWR produced less ROS.

It is worth noting that VWR induced several adaptations of skeletal muscle function independent of irradiation. However, these generalized benefits of exercise do not detract, but rather supplement, other benefits elicited specifically in the context of irradiation. A limitation of this study is that all analyses could not be made in the same muscle group. Since muscles involved in plantar flexion are generally more activated during VWR, it is possible that some effects are not detectable when evaluated in muscles that are only moderately recruited during VWR exercise. Another limitation of this study is that all studies used male mice. Female mice display both increased running activity [56] and muscle antioxidant activity [57]. Thus, adaptive responses of muscle to 1 month of VWR could be even greater in female mice. Previous studies reported that female survivors of childhood cancer are disproportionately affected by juvenile irradiation and present more frequently with sarcopenia [58]. Thus, females may experience both a greater response and benefit of exercise following juvenile irradiation. Future studies are needed to quantify the benefits of endurance exercise in juvenile female mice following radiation.

## Conclusions

Although 1 month of VWR did not prevent reduced muscle mass and fully restore raw force production, specific force production was rescued in both soleus and EDL muscles from irradiated mice. These results suggest that supplementing post-irradiation endurance exercise with an additional component of resistance training or interventions that target other juvenile radiation-related phenotypes such as muscle stem cell loss, persistent inflammation, and fibrosis [3–5] could be beneficial in stimulating muscle growth and hypertrophy, and thus, delay or possibly prevent the onset of sarcopenia. This study establishes that adaptations in Ca^2+^ handling, antioxidant response, and mitochondrial homeostasis following endurance exercise correlate with improved skeletal muscle functional outcomes post-juvenile irradiation treatment. These findings support the development of translational exercise treatment paradigms designed to maximize muscle function and improve the quality of life in the ever-growing population of juvenile cancer survivors.

## Supplementary Information

**Additional file 1.** Physiology ANOVA tables.

**Additional file 2.** Western blot.

**Additional file 3: Figure S1**. Exercise post-juvenile irradiation improves disrupted muscle contractile kinetics. EDL (left) and soleus (right) *ex vivo* physiology contractile kinetics. a, g) Absolute force frequency curve for 500 ms electrical stimulation. b, h) Specific force frequency curve for 500 ms electrical stimulation, normalized to physiologic cross-sectional area (P-CSA). c, i) Impulse (mN*sec) measurements taken as area under the curve for 500ms stimulation at 150Hz. d, j) Specific impulse (mN*sec/mm^2^) normalized to P-CSa. e, k) Rate of activation (mN/sec) measurements of excited muscle time-to-peak muscle contraction. f, l) Rate of relaxation (mN/sec) measurements of time to baseline following excitation stimulus cessation. n = 3-6 mice per condition. Two-way ANOVA with multiple comparisons, */# *p*<0.05, **/## *p*<0.01, ***/### *p*<0.001, ****/#### *p*<0.0001. Isolated asterisks denote ANOVA group effect of exercise. Isolated pound signs denote ANOVA group effect of radiation. Data displayed as mean +/- s.e.m.

**Additional file 4: Figure S2**. Endurance exercise does not increase irradiated muscle mass. EDL (left) and soleus (right). Raw muscle mass (mg) of a) EDL and b) soleus muscle from irradiated mouse contralateral (Rad CL) and irradiated (Rad RL) limbs, both exercised (running) and non-exercised (sedentary). n = 11 mice per condition. c, d) Lean muscle mass (muscle mass/body mass), from irradiated mouse contralateral (Rad CL) and irradiated (Rad RL) limbs, both exercised (running) and non-exercised (sedentary). n = 11 mice per condition. e) Myofiber cross-sectional area (CSA) from EDL and f) soleus muscle. Biological n=3 mice per condition, technical *n* value displayed directly on graph as number of myofibers measured per condition. Two-way ANOVA with multiple comparisons, * *p*<0.05, **/## *p*<0.01, ***/### *p*<0.001, ****/#### *p*<0.0001. Isolated asterisks denote ANOVA group effect of exercise. Isolated pound signs denote ANOVA group effect of radiation. Significant interaction between variables was observed in analysis of EDL CSA in e), *p*=0.033. Data displayed as mean +/- s.e.m.

**Additional file 5: Figure S3**. Exercise adapts calcium handling in irradiated muscle. a) TA whole muscle protein lysate immunoblot of non-irradiated (Rad CL) and irradiated (Rad RL) sedentary (Sed) and exercised (VWR) muscle. b) Quantification of protein levels of PMCA and c) NCX from immunoblot in a). d) TA whole muscle protein lysate immunoblot of non-irradiated (Rad CL) and irradiated (Rad RL) sedentary and exercised muscle. e) Quantification of protein levels of SERCA and f) MCU from immunoblot in d). All protein levels are normalized to GAPDH and non-irradiated (Rad CL) sedentary controls. n = 3 mice per condition except MCU, n = 6. Two-way ANOVA with multiple comparisons. * *p*<0.05, ** *p*<0.01, *** *p*<0.001. Isolated asterisks denote ANOVA group effect of exercise. Significant interaction between variables was observed in analysis of SERCA expression in d), *p*=0.017. Data displayed as mean +/- s.e.m.

## Data Availability

The datasets analyzed during the current study are available from the corresponding author upon reasonable request.

## Abbreviations

SARRP: Small animal radiation research platform
VWR: Voluntary wheel running
SR: Sarcoplasmic reticulum
EDL: Extensor digitorum longus muscle
ROS: Reactive oxygen species
RNS: Reactive nitrogen species
CSA: Cross-sectional area
ATP: Adenosine triphosphate
TA: Tibialis anterior muscle
P-CSA: Physiologic cross-sectional area
cDNA: Complementary DNA
RT-qPCR: Reverse transcriptase quantitative polymerase chain reaction
GAPDH: Glyceraldehyde-3-phosphate dehydrogenase
Dnm1l: Dynamin 1 like
Fis1: Fission, mitochondrial 1
Mfn1: Mitofusin 1
Pgc1α: Peroxisome proliferator-activated receptor gamma coactivator 1 alpha
RT: Room temperature
FDB: Flexor digitorum brevis muscle
3-NT: 3-Nitrotyrosine
MWF: Monday, Wednesday, and Friday
Rad RL: Irradiated mouse irradiated limb
Rad CL: Irradiated mouse, non-irradiated contralateral limb
0Gy CTL: Non-irradiated mouse control
CASQ1: Calsequestrin 1
SERCA: Sarco/endoplasmic reticulum calcium ATPase
CCD: Central core disease
MH: Malignant hyperthermia
MCU: Mitochondrial calcium uniporter
VDAC: Voltage-dependent anion channel
TAM: Tubular aggregate myopathy
RNAseq: RNA sequencing

## References

[1] Baskar R, Lee KA, Yeo R, Yeoh KW. Cancer and radiation therapy: current advances and future directions. Int J Med Sci. 2012;9. 10.7150/ijms.3635 PMC3298009 22408567

[2] Bachman JF, Chakkalakal JV. Insights into muscle stem cell dynamics during postnatal development. FEBS J. 2021. 10.1111/febs.15856 PMC9947813 33811430

[3] Paris ND, Kallenbach JG, Bachman JF, Blanc RS, Johnston CJ, Hernady E, et al. Chemoradiation impairs myofiber hypertrophic growth in a pediatric tumor model. Sci Rep. 2020;10(1). 10.1038/s41598-020-75913-w PMC7659015 33177579

[4] Bachman JF, Blanc RS, Paris ND, Kallenbach JG, Johnston CJ, Hernady E, et al. Radiation-induced damage to prepubertal Pax7+ skeletal muscle stem cells drives lifelong deficits in myofiber size and nuclear number. iScience. 2020;23(11). 10.1016/j.isci.2020.101760 PMC7674517 33241204

[5] Kallenbach JG, Bachman JF, Paris ND, Blanc RS, O’Connor T, Furati E, et al. Muscle-specific functional deficits and lifelong fibrosis in response to paediatric radiotherapy and tumour elimination. J Cachexia Sarcopenia Muscle. 2022. 10.1002/jcsm.12902 PMC8818600 34997696

[6] Ness KK, Baker KS, Dengel DR, Youngren N, Sibley S, Mertens AC, et al. Body composition, muscle strength deficits and mobility limitations in adult survivors of childhood acute lymphoblastic leukemia. Pediatr Blood Cancer. 2007;49(7). 10.1002/pbc.21091 17091482

[7] Ness KK, Mertens AC, Hudson MM, Wall MM, Leisenring WM, Oeffinger KC, et al. Limitations on physical performance and daily activities among long-term survivors of childhood cancer. Ann Intern Med. 2005;143(9). 10.7326/0003-4819-143-9-200511010-00007 16263886

[8] Ness KK, Armstrong GT, Kundu M, Wilson CL, Tchkonia T, Kirkland JL. Frailty in childhood cancer survivors. Cancer. 2015;121. 10.1002/cncr.29211 PMC4424063 25529481

[9] Paulino AC. Late effects of radiotherapy for pediatric extremity sarcomas. Int J Radiat Oncol Biol Phys. 2004;60(1). 10.1016/j.ijrobp.2004.02.001 15337565

[10] Gianfaldoni S, Gianfaldoni R, Wollina U, Lotti J, Tchernev G, Lotti T. An overview on radiotherapy: from its history to its current applications in dermatology. Open Access Maced J Med Sci. 2017;5(4). 10.3889/oamjms.2017.122 PMC5535674 28785349

[11] Quirós PM, Ramsay AJ, Sala D, Fernández-Vizarra E, Rodríguez F, Peinado JR, et al. Loss of mitochondrial protease OMA1 alters processing of the GTPase OPA1 and causes obesity and defective thermogenesis in mice. EMBO J. 2012;31(9). 10.1038/emboj.2012.70 PMC3343468 22433842

[12] Ratikan JA, Micewicz ED, Xie MW, Schaue D. Radiation takes its toll. Cancer Lett. 2015;368. 10.1016/j.canlet.2015.03.031 PMC4578968 25819030

[13] Yamamori T, Yasui H, Yamazumi M, Wada Y, Nakamura Y, Nakamura H, et al. Ionizing radiation induces mitochondrial reactive oxygen species production accompanied by upregulation of mitochondrial electron transport chain function and mitochondrial content under control of the cell cycle checkpoint. Free Radic Biol Med. 2012;53(2). 10.1016/j.freeradbiomed.2012.04.033 22580337

[14] Kam WWY, Banati RB. Effects of ionizing radiation on mitochondria. Free Radic Biol Med. 2013;65. 10.1016/j.freeradbiomed.2013.07.024 23892359

[15] Gaschler MM, Stockwell BR. Lipid peroxidation in cell death. Biochem Biophys Res Commun. 2017;482. 10.1016/j.bbrc.2016.10.086 PMC5319403 28212725

[16] Ferraro E, Giammarioli AM, Chiandotto S, Spoletini I, Rosano G. Exercise-induced skeletal muscle remodeling and metabolic adaptation: redox signaling and role of autophagy. Antioxid Redox Signal. 2014;21(1). 10.1089/ars.2013.5773 PMC4048572 24450966

[17] Yukawa O, Nakazawa T. Radiation-induced lipid peroxidation and membrane-bound enzymes in liver microsomes. Int J Radiat Biol. 1980;37(5). 6968297

[18] Robinson MM, Dasari S, Konopka AR, Johnson ML, Manjunatha S, Esponda RR, et al. Enhanced protein translation underlies improved metabolic and physical adaptations to different exercise training modes in young and old humans. Cell Metab. 2017;25(3). 10.1016/j.cmet.2017.02.009 PMC5423095 28273480

[19] Michelucci A, Boncompagni S, Pietrangelo L, García-Castañeda M, Takano T, Malik S, et al. Transverse tubule remodeling enhances orai1-dependent Ca2+ entry in skeletal muscle. eLife. 2019;8. 10.7554/eLife.47576 PMC6837846 31657717

[20] Boncompagni S, Rossi AE, Micaroni M, Beznoussenko GV, Polishchuk RS, Dirksen RT, et al. Mitochondria are linked to calcium stores in striated muscle by developmentally regulated tethering structures. Mol Biol Cell. 2009;20(3). 10.1091/mbc.E08-07-0783 PMC2633377 19037102

[21] Bachman JF, Klose A, Liu W, Paris ND, Blanc RS, Schmalz M, et al. Prepubertal skeletal muscle growth requires pax7-expressing satellite cell-derived myonuclear contribution. Development (Cambridge). 2018;145(20). 10.1242/dev.167197 PMC6215399 30305290

[22] Pietrangelo L, Michelucci A, Ambrogini P, Sartini S, Guarnier FA, Fusella A, et al. Muscle activity prevents the uncoupling of mitochondria from Ca 2+ release units induced by ageing and disuse. Arch Biochem Biophys. 2019;663. 10.1016/j.abb.2018.12.017 PMC6377823 30578752

[23] Powers SK, Jackson MJ. Exercise-induced oxidative stress: cellular mechanisms and impact on muscle force production. Physiol Rev. 2008;88. 10.1152/physrev.00031.2007 PMC2909187 18923182

[24] Liu W, Klose A, Forman S, Paris ND, Wei-LaPierre L, Cortés-Lopéz M, et al. Loss of adult skeletal muscle stem cells drives age-related neuromuscular junction degeneration. eLife. 2017;6. 10.7554/eLife.26464 PMC5462534 28583253

[25] Liu W, Wei-LaPierre L, Klose A, Dirksen RT, Chakkalakal JV. Inducible depletion of adult skeletal muscle stem cells impairs the regeneration of neuromuscular junctions. eLife. 2015;4(AUGUST2015). 10.7554/eLife.09221 PMC4579298 26312504

[26] Hakim CH, Wasala NB, Duan D. Evaluation of muscle function of the extensor digitorum longus muscle ex vivo and tibialis anterior muscle in situ in mice. J Vis Exp. 2013;(72). 10.3791/50183 PMC3601038 23426237

[27] Lanner JT, Georgiou DK, Dagnino-Acosta A, Ainbinder A, Cheng Q, Joshi AD, et al. AICAR prevents heat-induced sudden death in RyR1 mutant mice independent of AMPK activation. Nat Med. 2012;18(2). 10.1038/nm.2598 PMC3274651 22231556

[28] Lee CS, Hanna AD, Wang H, Dagnino-Acosta A, Joshi AD, Knoblauch M, et al. A chemical chaperone improves muscle function in mice with a RyR1 mutation. Nature. Communications. 2017;8. 10.1038/ncomms14659 PMC5376670 28337975

[29] Michelucci A, García-Castañeda M, Boncompagni S, Dirksen RT. Role of STIM1/ORAI1-mediated store-operated Ca2+ entry in skeletal muscle physiology and disease. Cell Calcium. 2018;76. 10.1016/j.ceca.2018.10.004 PMC6290926 30414508

[30] Coronado R, Ahern CA, Sheridan DC, Cheng W, Carbonneau L, Bhattacharya D. Functional equivalence of dihydropyridine receptor alpha1S and beta1a subunits in triggering excitation-contraction coupling in skeletal muscle. Biol Res. 2004;37. 10.4067/s0716-97602004000400010 15709683

[31] Durham WJ, Aracena-Parks P, Long C, Rossi AE, Goonasekera SA, Boncompagni S, et al. RyR1 S-nitrosylation underlies environmental heat stroke and sudden death in Y522S RyR1 knockin mice. Cell. 2008;133(1). 10.1016/j.cell.2008.02.042 PMC2366094 18394989

[32] Canato M, Capitanio P, Cancellara L, Leanza L, Raffaello A, Reane DV, et al. Excessive accumulation of Ca2 + in mitochondria of Y522S-RYR1 knock-in mice: a link between leak from the sarcoplasmic reticulum and altered redox state. Front Physiol. 2019;10. 10.3389/fphys.2019.01142 PMC6755340 31607937

[33] Avila G, Lee EH, Perez CF, Allen PD, Dirksen RT. FKBP12 binding to RyR1 modulates excitation-contraction coupling in mouse skeletal myotubes. J Biol Chem. 2003;278(25). 10.1074/jbc.M205866200 12704193

[34] Boncompagni S, Loy RE, Dirksen RT, Franzini-Armstrong C. The I4895T mutation in the type 1 ryanodine receptor induces fiber-type specific alterations in skeletal muscle that mimic premature aging. Aging Cell. 2010;9(6). 10.1111/j.1474-9726.2010.00623.x PMC2980556 20961389

[35] de Lisio M, Kaczor JJ, Phan N, Tarnopolsky MA, Boreham DR, Parise G. Exercise training enhances the skeletal muscle response to radiation-induced oxidative stress. Muscle Nerve. 2011;43(1). 10.1002/mus.21797 21171096

[36] Purbey PK, Scumpia PO, Kim PJ, Tong AJ, Iwamoto KS, McBride WH, et al. Defined sensing mechanisms and signaling pathways contribute to the global inflammatory gene expression output elicited by ionizing radiation. Immunity. 2017;47(3). 10.1016/j.immuni.2017.08.017 PMC5661954 28930658

[37] Anuranjani BM. Concerted action of Nrf2-ARE pathway, MRN complex, HMGB1 and inflammatory cytokines - implication in modification of radiation damage. Redox Biol. 2014;2. 10.1016/j.redox.2014.02.008 PMC4085347 25009785

[38] McBride WH, Schaue D. Radiation-induced tissue damage and response. J Pathol. 2020;250. 10.1002/path.5389 PMC7216989 31990369

[39] Nilsson MI, Bourgeois JM, Nederveen JP, Leite MR, Hettinga BP, Bujak AL, et al. Lifelong aerobic exercise protects against inflammaging and cancer. PLoS One. 2019;14(1). 10.1371/journal.pone.0210863 PMC6347267 30682077

[40] Michelucci A, Paolini C, Boncompagni S, Canato M, Reggiani C, Protasi F. Strenuous exercise triggers a life-threatening response in mice susceptible to malignant hyperthermia. FASEB J. 2017;31(8). 10.1096/fj.201601292R PMC5503704 28465322

[41] Robinson KM, Janes MS, Pehar M, Monette JS, Ross MF, Hagen TM, et al. Selective fluorescent imaging of superoxide in vivo using ethidium-based probes. Proc Natl Acad Sci U S A. 2006;103(41). 10.1073/pnas.0601945103 PMC1586181 17015830

[42] Qaisar R, Bhaskaran S, van Remmen H. Muscle fiber type diversification during exercise and regeneration. Free Radic Biol Med. 2016;98. 10.1016/j.freeradbiomed.2016.03.025 27032709

[43] Jackson JR, Kirby TJ, Fry CS, Cooper RL, McCarthy JJ, Peterson CA, et al. Reduced voluntary running performance is associated with impaired coordination as a result of muscle satellite cell depletion in adult mice. Skelet Muscle. 2015;5(1). 10.1186/s13395-015-0065-3 PMC4647638 26579218

[44] Vinel C, Lukjanenko L, Batut A, Deleruyelle S, Pradère JP, le Gonidec S, et al. The exerkine apelin reverses age-associated sarcopenia. Nat Med. 2018;24(9). 10.1038/s41591-018-0131-6 30061698

[45] Manzanares G, Brito-Da-Silva G, Gandra PG. Voluntary wheel running: patterns and physiological effects in mice. Braz J Med Biol Res. 2019;52(1). 10.1590/1414-431X20187830 PMC6301263 30539969

[46] Brookes PS, Yoon Y, Robotham JL, Anders MW, Sheu SS. Calcium, ATP, and ROS: a mitochondrial love-hate triangle. Am J Physiol Cell Physiol. 2004;287. 10.1152/ajpcell.00139.2004 15355853

[47] Shishmarev D. Excitation-contraction coupling in skeletal muscle: recent progress and unanswered questions. Biophys Rev. 2020;12. 10.1007/s12551-020-00610-x PMC7040155 31950344

[48] Michelucci A, Liang C, Protasi F, Dirksen RT. Altered Ca2+ handling and oxidative stress underlie mitochondrial damage and skeletal muscle dysfunction in aging and disease. Metabolites. 2021;11. 10.3390/metabo11070424 PMC8304741 34203260

[49] Perez CF, Eltit JM, Lopez JR, Bodnár D, Dulhunty AF, Aditya S, et al. Functional and structural characterization of a novel malignant hyperthermia-susceptible variant of DHPR-β1a subunit (CACNB1). Am J Physiol Cell Physiol. 2018;314(3). 10.1152/ajpcell.00187.2017 PMC6335014 29212769

[50] Boncompagni S, Michelucci A, Pietrangelo L, Dirksen RT, Protasi F. Exercise-dependent formation of new junctions that promote STIM1-Orai1 assembly in skeletal muscle. Sci Rep. 2017;7(1). 10.1038/s41598-017-14134-0 PMC5660245 29079778

[51] Rossi AE, Boncompagni S, Dirksen RT. Sarcoplasmic reticulum-mitochondrial symbiosis: bidirectional signaling in skeletal muscle. Exerc Sport Sci Rev. 2009;37. 10.1097/JES.0b013e3181911fa4 PMC2740713 19098522

[52] Brini M, Carafoli E. The plasma membrane Ca2+ ATPase and the plasma membrane sodium calcium exchanger cooperate in the regulation of cell calcium. Cold Spring Harb Perspect Biol. 2011;3(2). 10.1101/cshperspect.a004168 PMC3039526 21421919

[53] Azzam EI, Jay-Gerin JP, Pain D. Ionizing radiation-induced metabolic oxidative stress and prolonged cell injury. Cancer Lett. 2012;327. 10.1016/j.canlet.2011.12.012 PMC3980444 22182453

[54] Ivarsson N, Mikael Mattsson C, Cheng AJ, Bruton JD, Ekblom B, Lanner JT, et al. SR Ca2+ leak in skeletal muscle fibers acts as an intracellular signal to increase fatigue resistance. J Gen Physiol. 2019;151(4). 10.1085/jgp.201812152 PMC6445590 30635368

[55] Zanou N, Dridi H, Reiken S, Imamura de Lima T, Donnelly C, de Marchi U (2021). Acute RyR1 Ca2+ leak enhances NADH-linked mitochondrial respiratory capacity. Nature. Communications.

[56] Claghorn GC, Thompson Z, Wi K, Van L, Garland T. Caffeine stimulates voluntary wheel running in mice without increasing aerobic capacity. Physiol Behav. 2017;170. 10.1016/j.physbeh.2016.12.031 28039074

[57] Michelucci A, Boncompagni S, Canato M, Reggiani C, Protasi F. Estrogens protect calsequestrin-1 knockout mice from lethal hyperthermic episodes by reducing oxidative stress in muscle. Oxidative Med Cell Longev. 2017;2017. 10.1155/2017/6936897 PMC5610815 29062464

[58] McCastlain K, Howell CR, Welsh CE, Wang Z, Wilson CL, Mulder HL, et al. The association of mitochondrial copy number with sarcopenia in adult survivors of childhood cancer. J Natl Cancer Inst. 2021;113(11). 10.1093/jnci/djab084 PMC8562958 33871611

