# Supplementary material for: Endurance exercise attenuates juvenile irradiation-induced skeletal muscle functional decline and mitochondrial stress
Source: Skelet Muscle. 2022 Apr 12;12:8. doi: 10.1186/s13395-022-00291-y (PMC9004104; doi:10.1186/s13395-022-00291-y)
Supplement: Supplementary file 2 — Additional file 2. Western blot. [file 13395_2022_291_MOESM2_ESM.pptx]

## Slide 1
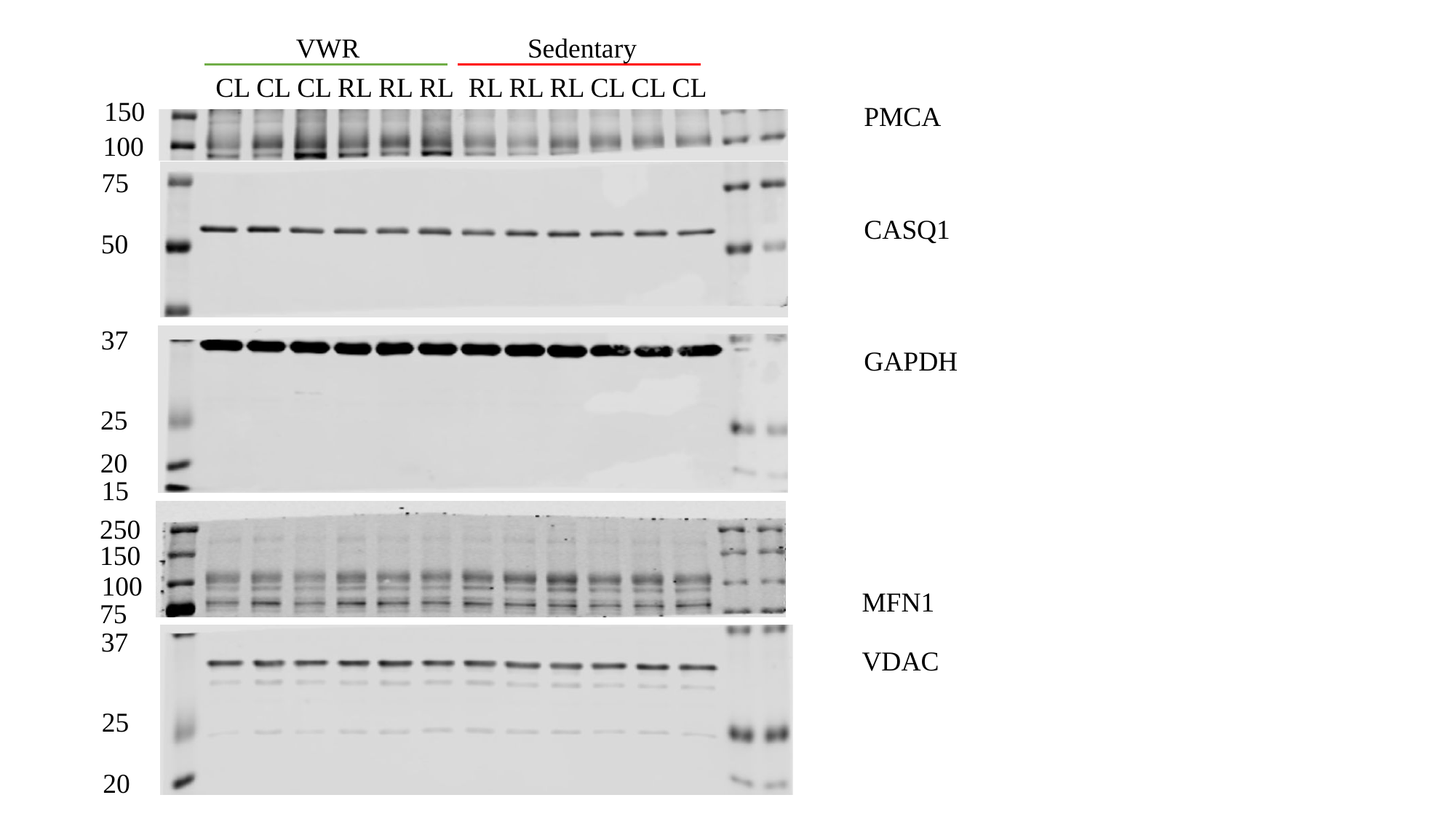

Sedentary
VWR
CL CL CL RL RL RL
RL RL RL CL CL CL
150
PMCA
100
75
CASQ1
50
37
GAPDH
25
20
15
250
150
100
MFN1
75
37
VDAC
25
20

## Slide 2
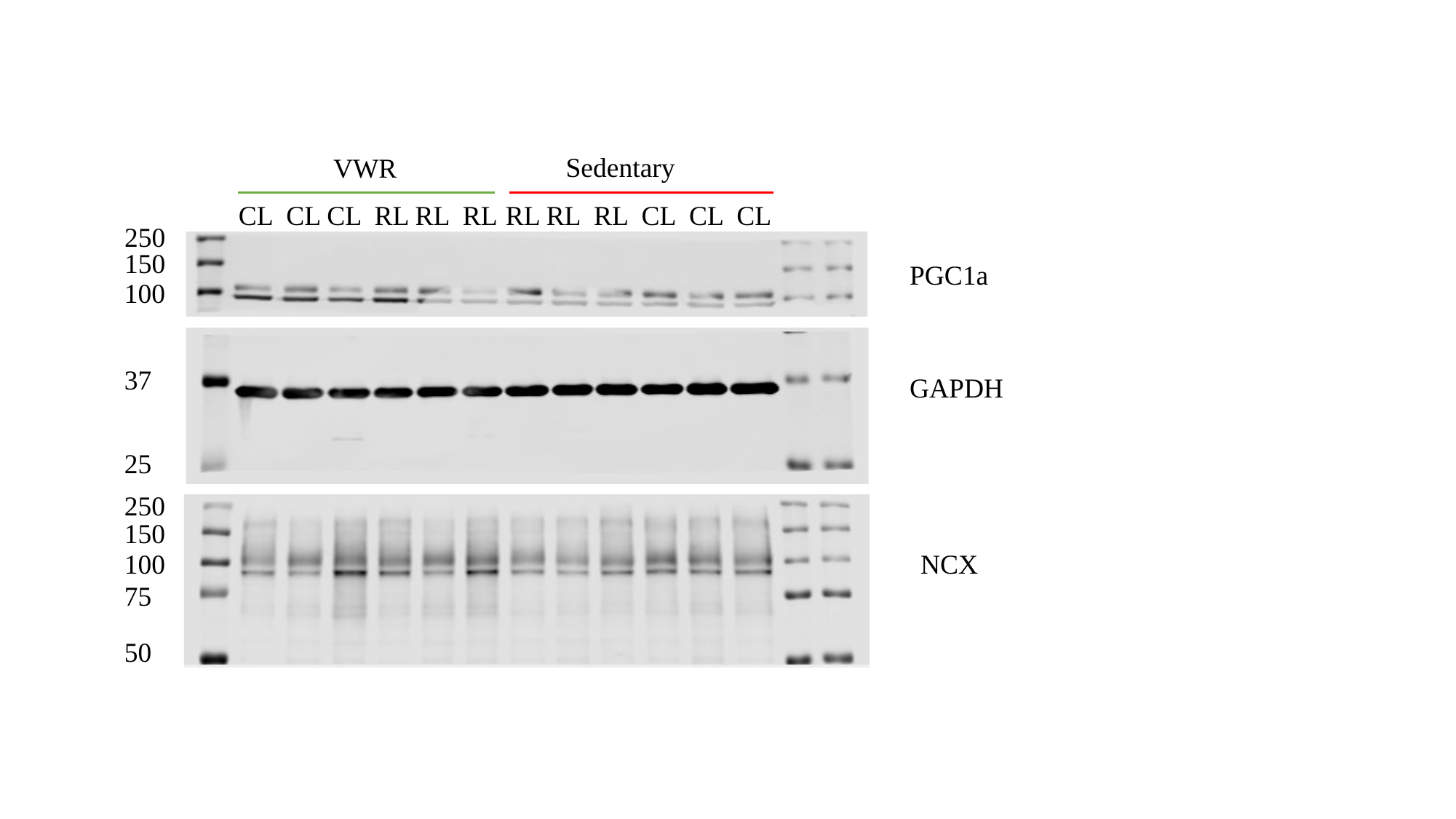

Sedentary
VWR
CL CL CL RL RL RL
RL RL RL CL CL CL
250
150
PGC1a
100
37
GAPDH
25
250
150
100
NCX
75
50

## Slide 3
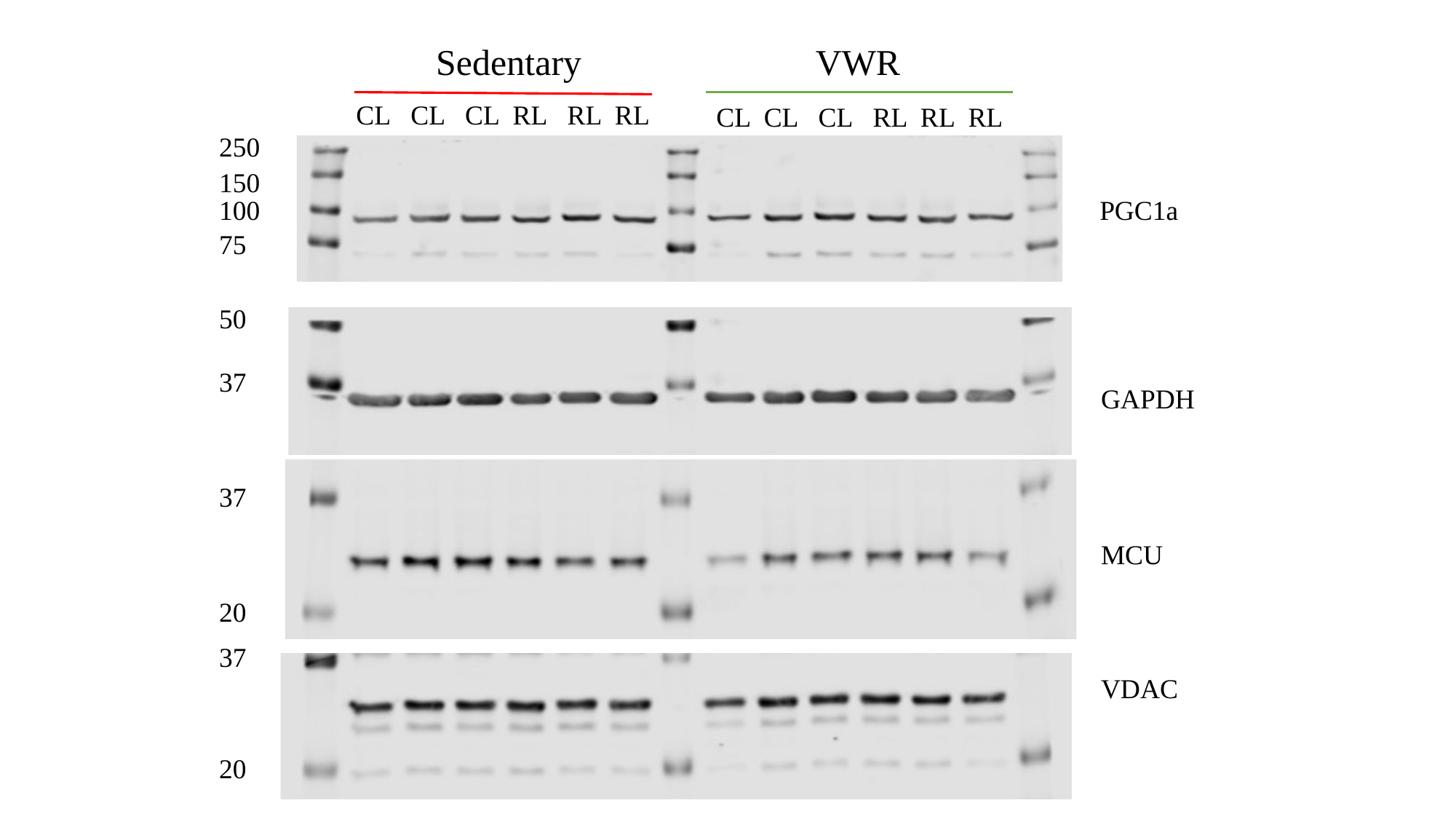

Sedentary
VWR
 CL CL CL RL RL RL
CL CL CL RL RL RL
250
150
100
PGC1a
75
50
37
GAPDH
37
MCU
20
37
VDAC
20

## Slide 4
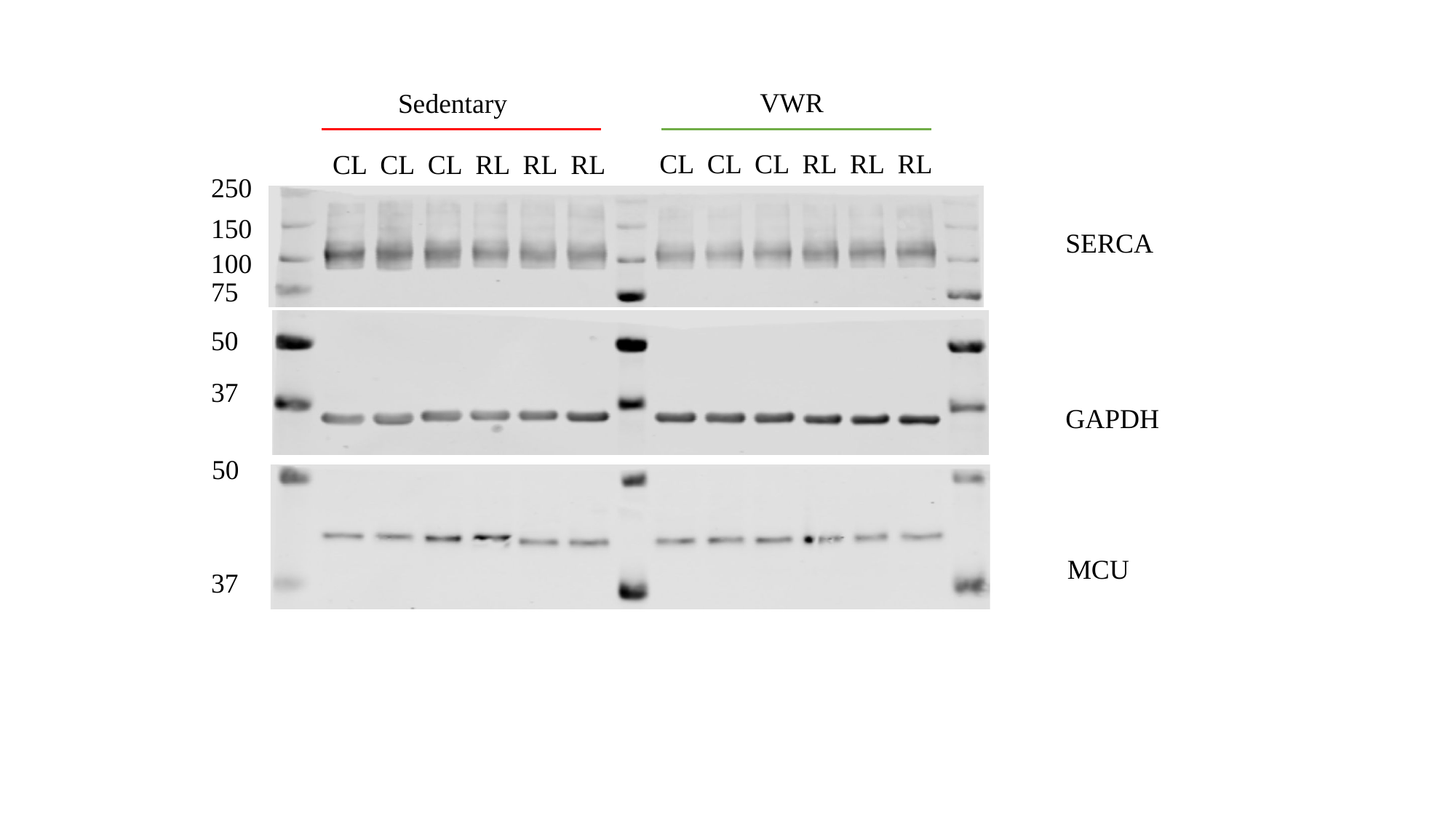

VWR
Sedentary
CL CL CL RL RL RL
CL CL CL RL RL RL
250
150
SERCA
100
75
50
37
GAPDH
50
MCU
37

## Slide 5
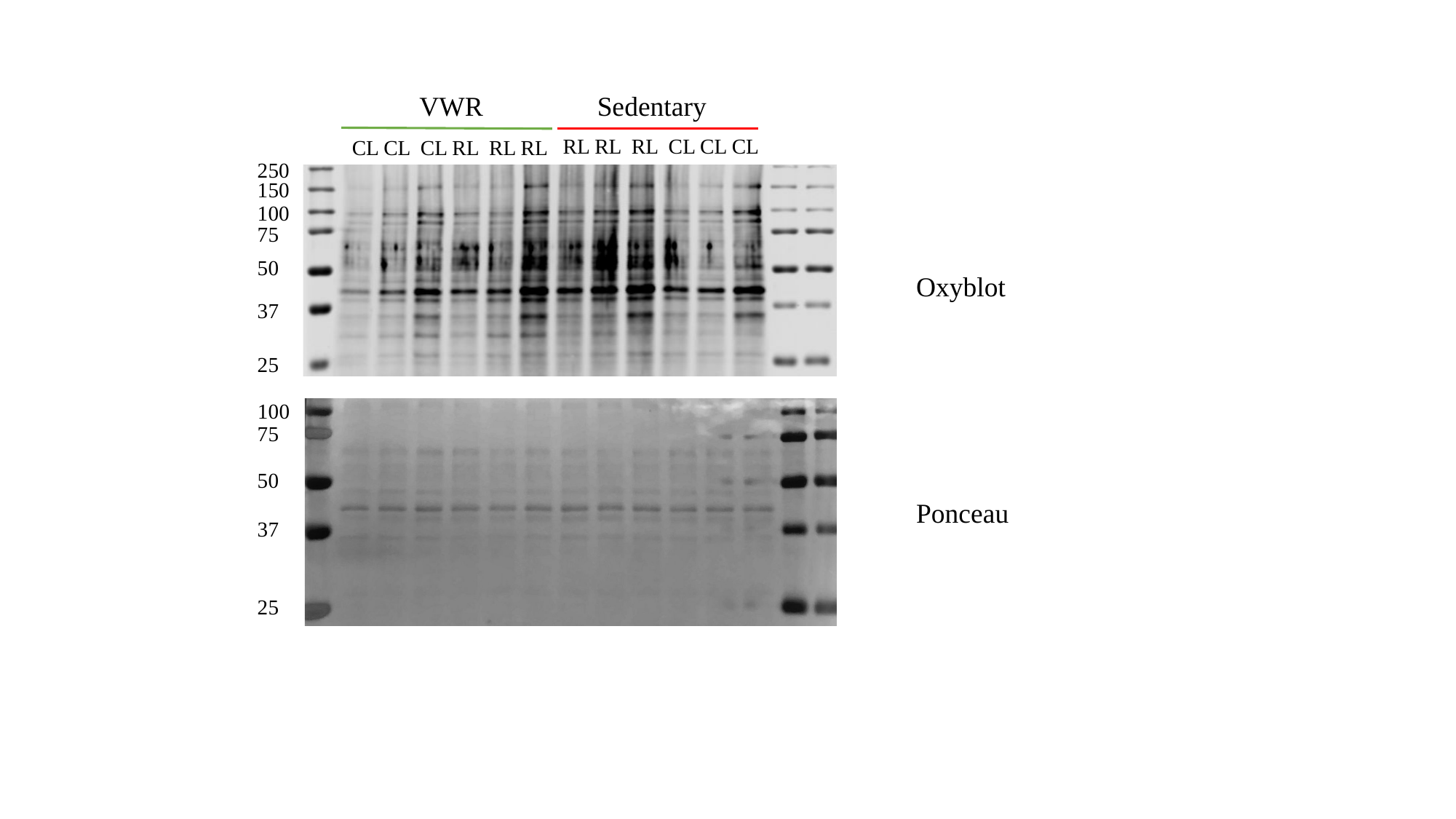

Sedentary
VWR
RL RL RL CL CL CL
CL CL CL RL RL RL
250
150
100
75
50
Oxyblot
37
25
100
75
50
Ponceau
37
25

## Slide 6
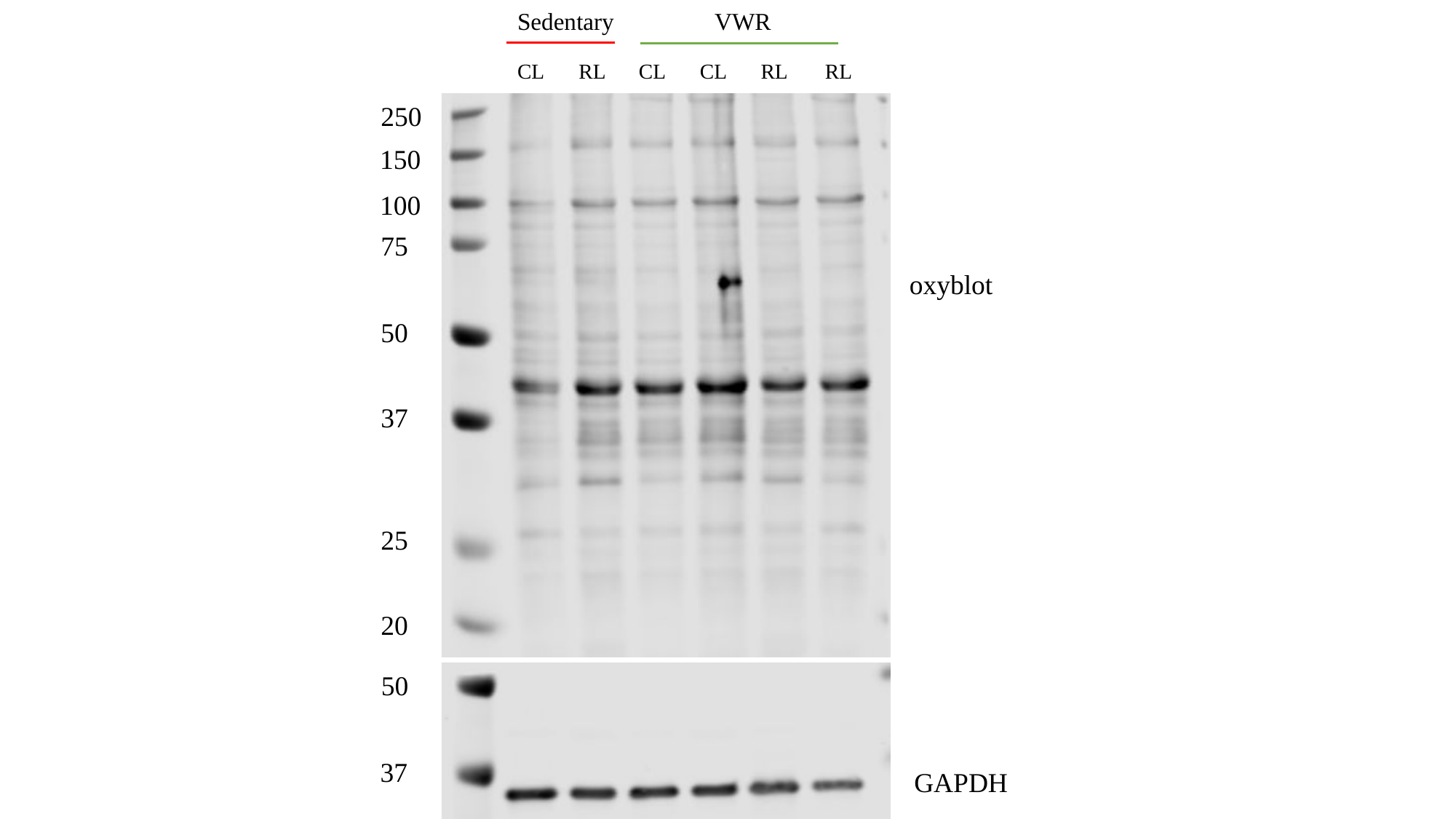

Sedentary
VWR
CL
RL
CL
CL
RL
RL
250
150
100
75
oxyblot
50
37
25
20
50
37
GAPDH

## Slide 7
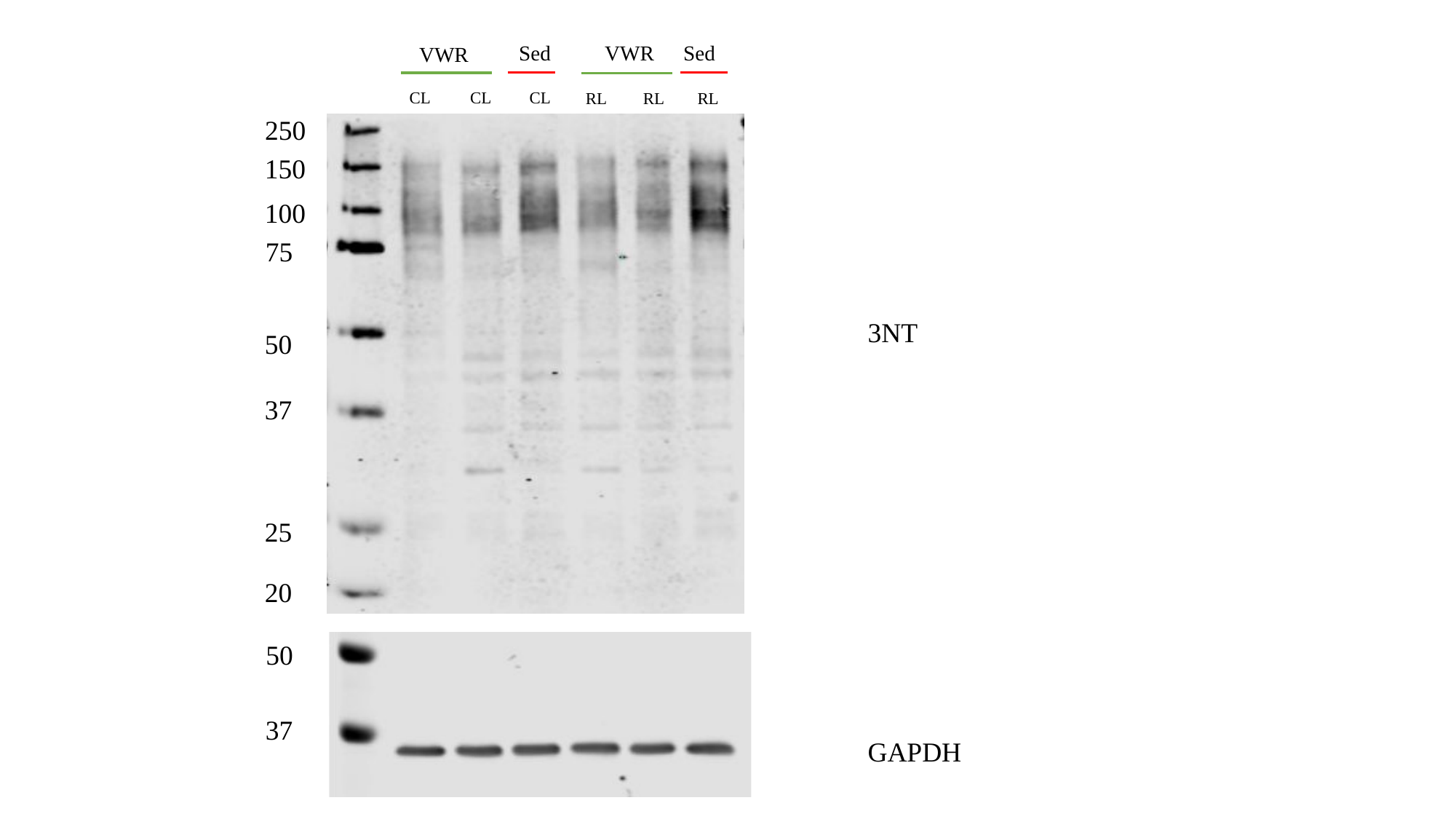

Sed
VWR
Sed
VWR
CL
CL
CL
RL
RL
RL
250
150
100
75
3NT
50
37
25
20
50
37
GAPDH

## Slide 8
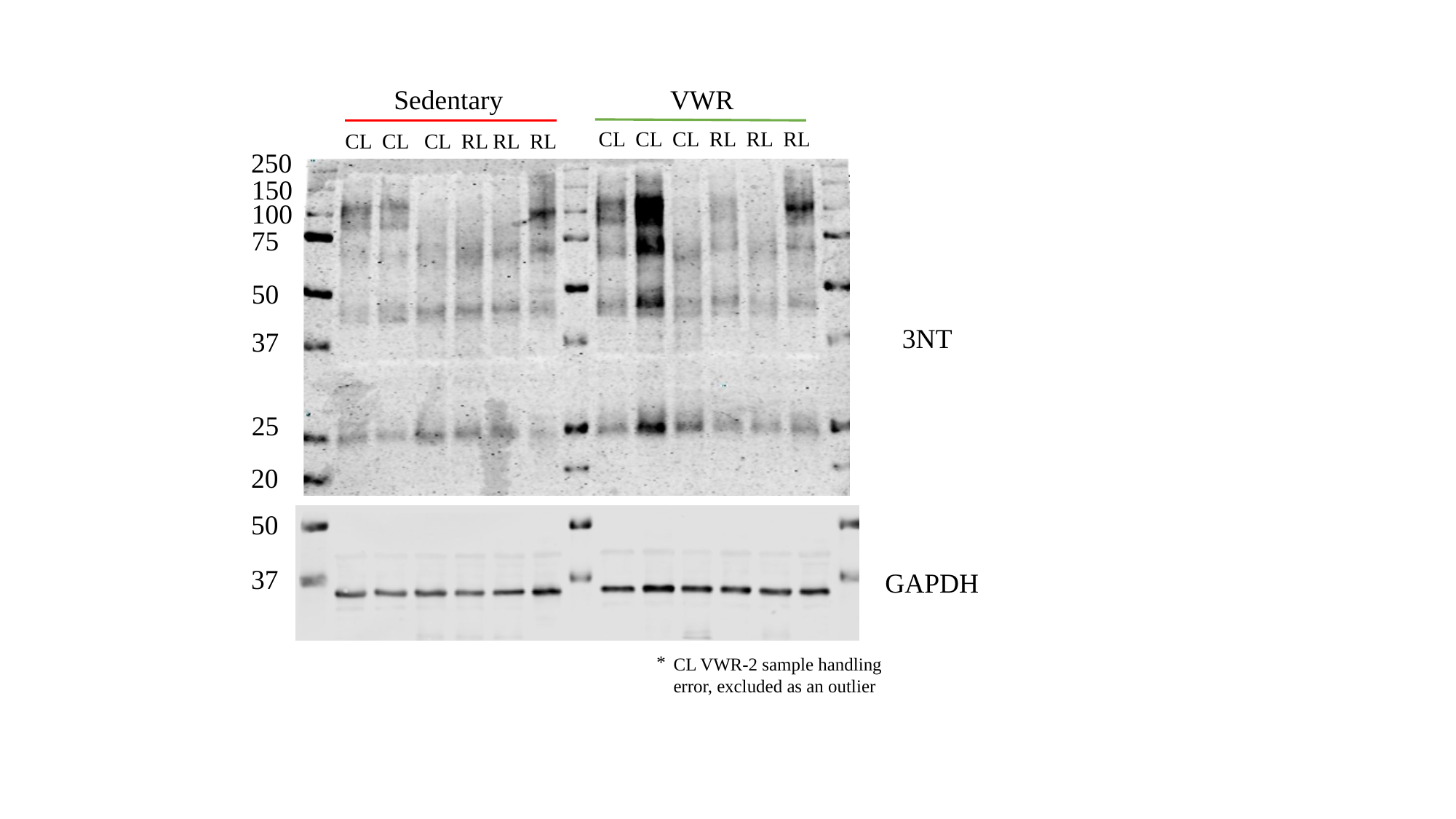

VWR
Sedentary
 CL CL CL RL RL RL
CL CL CL RL RL RL
250
150
100
75
50
3NT
37
25
20
50
37
GAPDH
*
CL VWR-2 sample handling error, excluded as an outlier
