# Supplementary figures and images for: Endurance exercise attenuates juvenile irradiation-induced skeletal muscle functional decline and mitochondrial stress
Source: Skelet Muscle. 2022 Apr 12;12:8. doi: 10.1186/s13395-022-00291-y (PMC9004104; doi:10.1186/s13395-022-00291-y)

## Slide 1
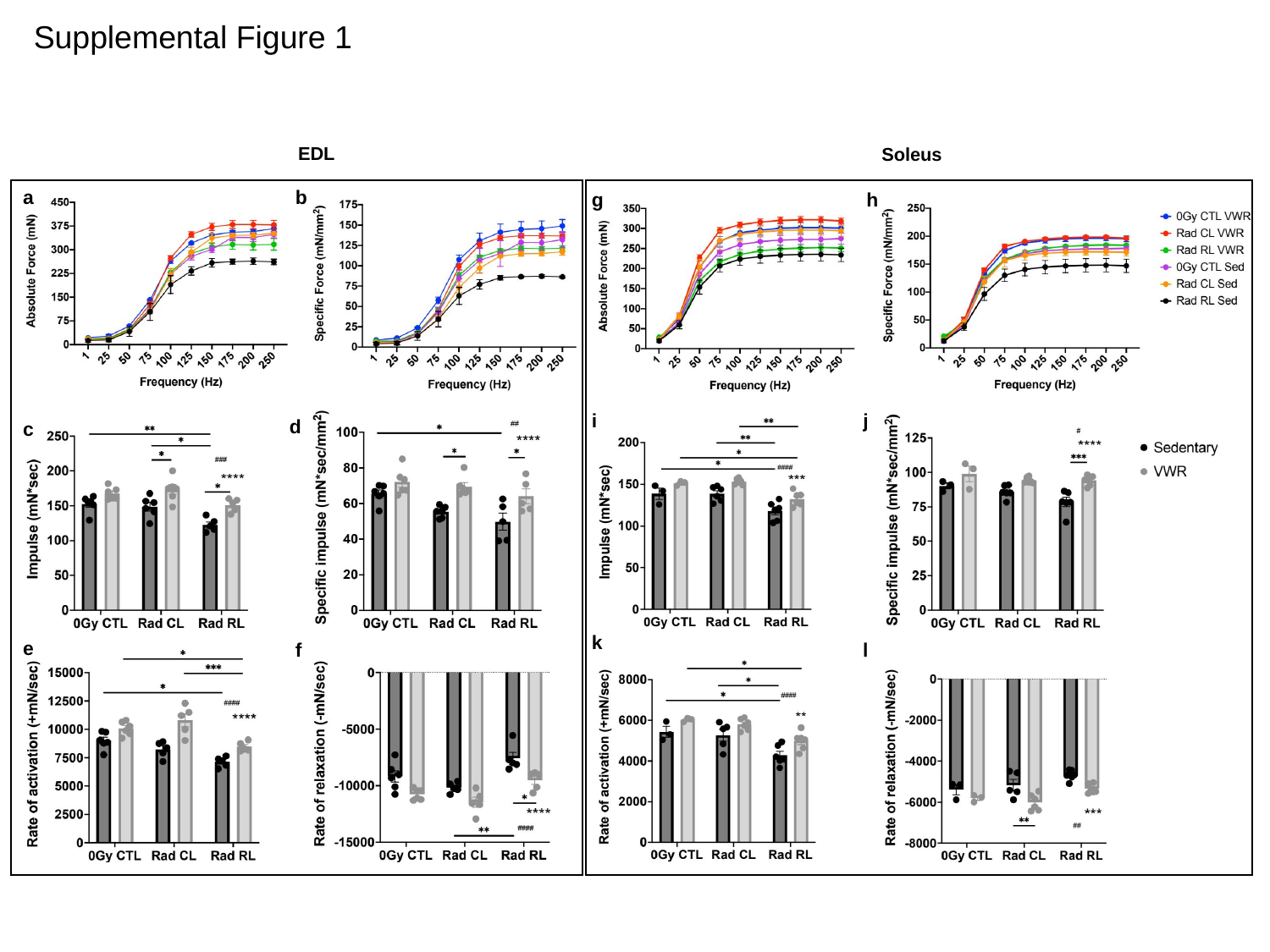

Supplemental Figure 1
EDL
Soleus
a
b
g
h
j
i
d
c
k
e
f
l

Supplement: Supplementary file 3 — Additional file 3: Figure S1. Exercise post-juvenile irradiation improves disrupted muscle contractile kinetics. EDL (left) and soleus (right) ex vivo physiology contractile kinetics. a, g) Absolute force frequency curve for 500 ms electrical stimulation. b, h) Specific force frequency curve for 500 ms electrical stimulation, normalized to physiologic cross-sectional area (P-CSA). c, i) Impulse (mN*sec) measurements taken as area under the curve for 500ms stimulation at 150Hz. d, j) Specific impulse (mN*sec/mm2) normalized to P-CSa. e, k) Rate of activation (mN/sec) measurements of excited muscle time-to-peak muscle contraction. f, l) Rate of relaxation (mN/sec) measurements of time to baseline following excitation stimulus cessation. n = 3-6 mice per condition. Two-way ANOVA with multiple comparisons, */# p<0.05, **/## p<0.01, ***/### p<0.001, ****/#### p<0.0001. Isolated asterisks denote ANOVA group effect of exercise. Isolated pound signs denote ANOVA group effect of radiation. Data displayed as mean +/- s.e.m. [file 13395_2022_291_MOESM3_ESM.pptx]

## Slide 1
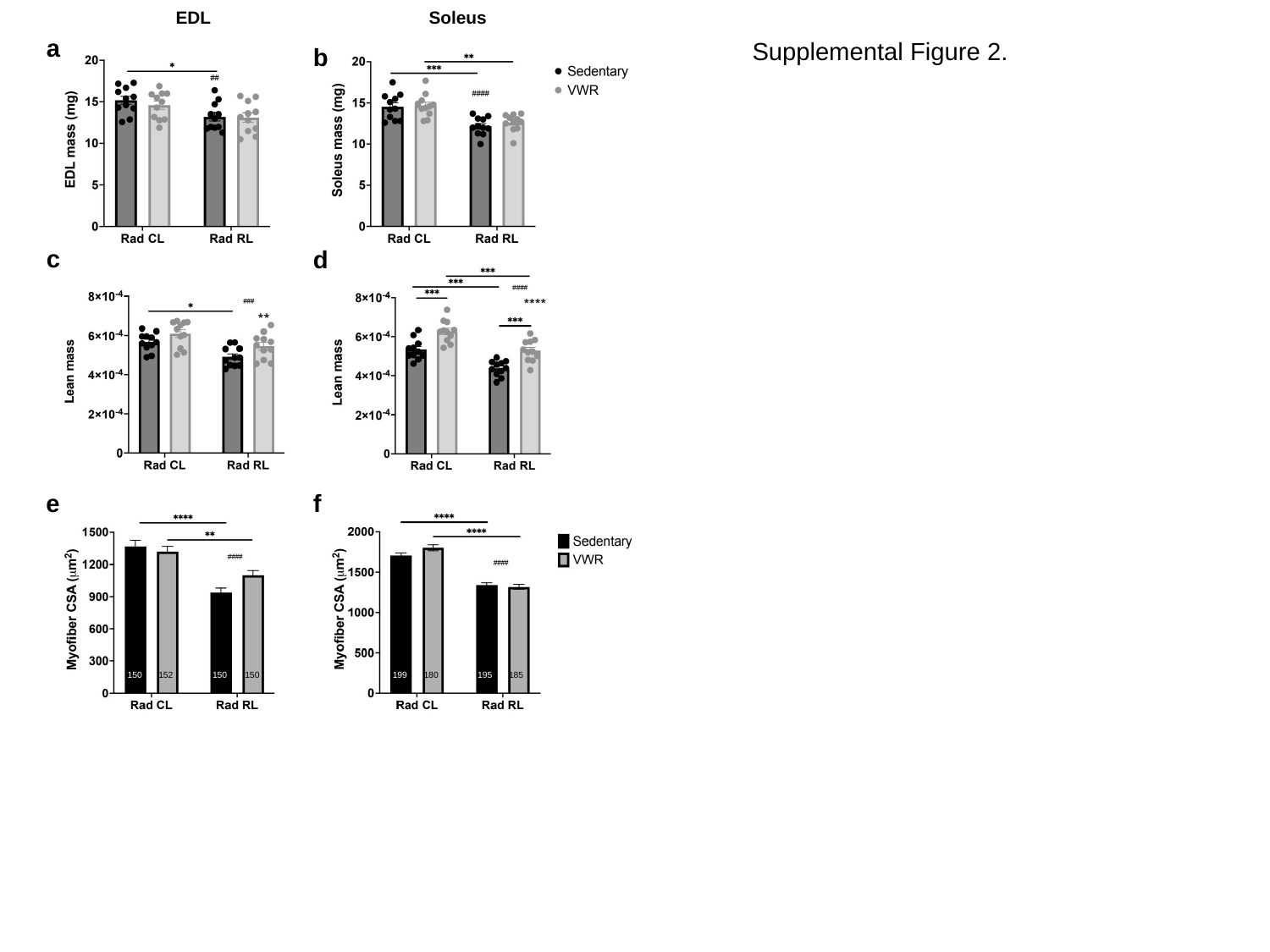

EDL
Soleus
a
Supplemental Figure 2.
b
c
d
f
e
 199
180
185
152
 195
 150
150
150

Supplement: Supplementary file 4 — Additional file 4: Figure S2. Endurance exercise does not increase irradiated muscle mass. EDL (left) and soleus (right). Raw muscle mass (mg) of a) EDL and b) soleus muscle from irradiated mouse contralateral (Rad CL) and irradiated (Rad RL) limbs, both exercised (running) and non-exercised (sedentary). n = 11 mice per condition. c, d) Lean muscle mass (muscle mass/body mass), from irradiated mouse contralateral (Rad CL) and irradiated (Rad RL) limbs, both exercised (running) and non-exercised (sedentary). n = 11 mice per condition. e) Myofiber cross-sectional area (CSA) from EDL and f) soleus muscle. Biological n=3 mice per condition, technical n value displayed directly on graph as number of myofibers measured per condition. Two-way ANOVA with multiple comparisons, * p<0.05, **/## p<0.01, ***/### p<0.001, ****/#### p<0.0001. Isolated asterisks denote ANOVA group effect of exercise. Isolated pound signs denote ANOVA group effect of radiation. Significant interaction between variables was observed in analysis of EDL CSA in e), p=0.033. Data displayed as mean +/- s.e.m. [file 13395_2022_291_MOESM4_ESM.pptx]
