# Supplementary material for: Endurance exercise attenuates juvenile irradiation-induced skeletal muscle functional decline and mitochondrial stress
Source: Skelet Muscle. 2022 Apr 12;12:8. doi: 10.1186/s13395-022-00291-y (PMC9004104; doi:10.1186/s13395-022-00291-y)
Supplement: Supplementary file 5 — Additional file 5: Figure S3. Exercise adapts calcium handling in irradiated muscle. a) TA whole muscle protein lysate immunoblot of non-irradiated (Rad CL) and irradiated (Rad RL) sedentary (Sed) and exercised (VWR) muscle. b) Quantification of protein levels of PMCA and c) NCX from immunoblot in a). d) TA whole muscle protein lysate immunoblot of non-irradiated (Rad CL) and irradiated (Rad RL) sedentary and exercised muscle. e) Quantification of protein levels of SERCA and f) MCU from immunoblot in d). All protein levels are normalized to GAPDH and non-irradiated (Rad CL) sedentary controls. n = 3 mice per condition except MCU, n = 6. Two-way ANOVA with multiple comparisons. * p<0.05, ** p<0.01, *** p<0.001. Isolated asterisks denote ANOVA group effect of exercise. Significant interaction between variables was observed in analysis of SERCA expression in d), p=0.017. Data displayed as mean +/- s.e.m. [file 13395_2022_291_MOESM5_ESM.pptx]

## Slide 1
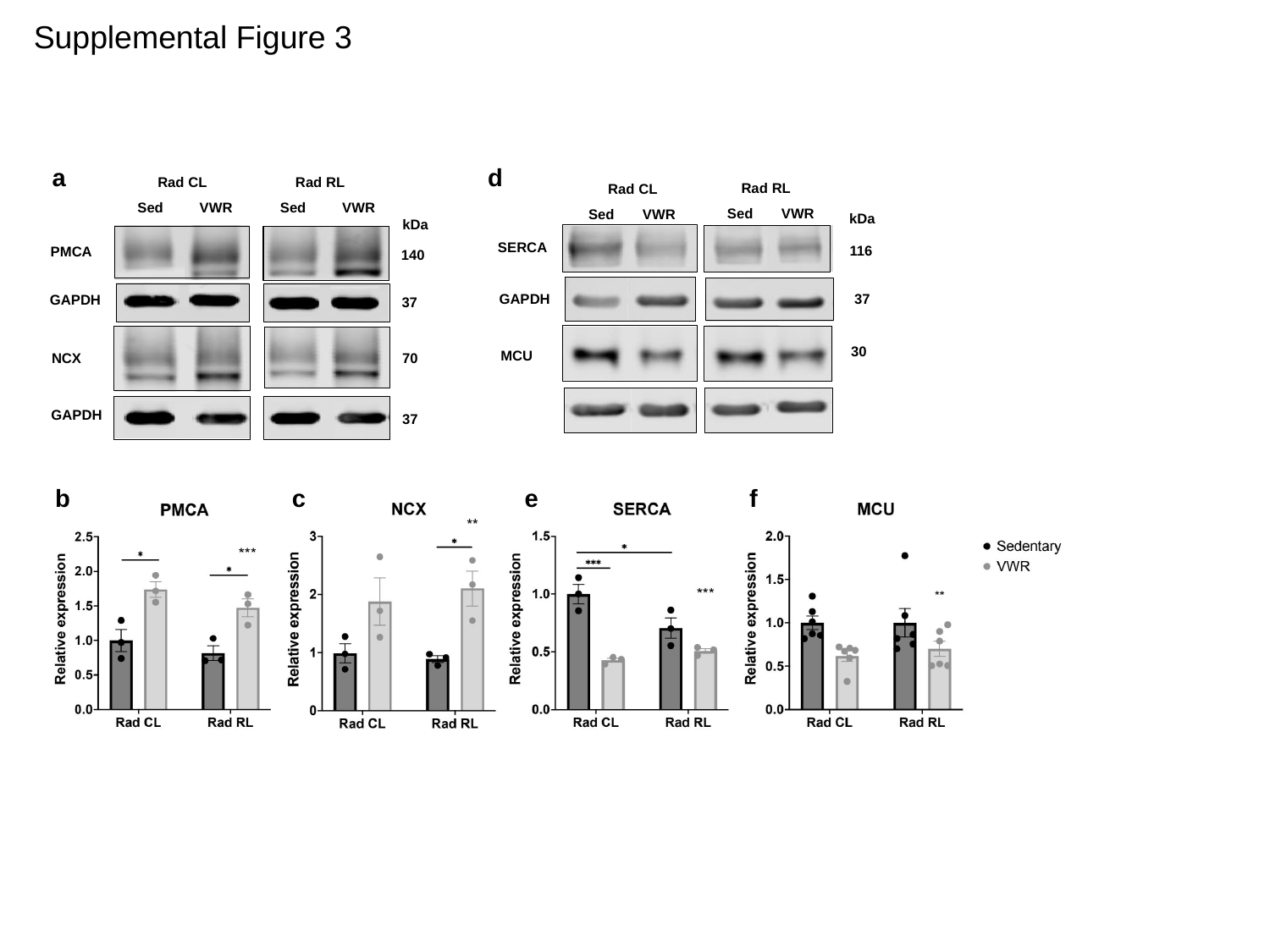

Supplemental Figure 3
a
d
Rad RL
Rad CL
Rad RL
Rad CL
 Sed VWR
 Sed VWR
Sed VWR
Sed VWR
kDa
kDa
SERCA
116
PMCA
140
37
GAPDH
GAPDH
37
30
MCU
NCX
70
GAPDH
37
e
f
b
c
